# Quorum Quenching Revisited—From Signal Decays to Signalling Confusion

**DOI:** 10.3390/s120404661

**Published:** 2012-04-10

**Authors:** Kar-Wai Hong, Chong-Lek Koh, Choon-Kook Sam, Wai-Fong Yin, Kok-Gan Chan

**Affiliations:** 1 Division of Genetics and Molecular Biology, Institute of Biological Sciences, Faculty of Science, University of Malaya, Kuala Lumpur 50603, Malaysia; 2 Natural Sciences and Science Education AG, National Institute of Education, Nanyang Technological University, 1 Nanyang Walk, Singapore 637616, Singapore

**Keywords:** acylase, anti-infective, anti-biofouling, aquaculture, lactonase, *N*-acylhomoserine lactone, paraoxonase, oxidoreductase, quorum sensing, quorum quenching resistance, signalling confusion

## Abstract

In a polymicrobial community, while some bacteria are communicating with neighboring cells (quorum sensing), others are interrupting the communication (quorum quenching), thus creating a constant arms race between intercellular communication. In the past decade, numerous quorum quenching enzymes have been found and initially thought to inactivate the signalling molecules. Though this is widely accepted, the actual roles of these quorum quenching enzymes are now being uncovered. Recent evidence extends the role of quorum quenching to detoxification or metabolism of signalling molecules as food and energy source; this includes “signalling confusion”, a term coined in this paper to refer to the phenomenon of non-destructive modification of signalling molecules. While quorum quenching has been explored as a novel anti-infective therapy targeting, quorum sensing evidence begins to show the development of resistance against quorum quenching.

## 1. Introduction

In the early 1970s, the belief that the individual cells in a bacterial population function as autonomous units has been supplanted with the in-depth understanding of cell-to-cell communication, which is also known as quorum sensing (QS). QS is prevalent throughout the Eubacteria domain, allowing bacteria to regulate gene expression in a population-dependent manner, in response to the concentration of diffusible chemical signals produced and released into the local environment by themselves or other bacteria, either of the same or different species, thus allowing synchronized bacterial behaviors acting in unison [1–3].

Bacteria appear to be linguistic and several QS signals have been identified, ranging from low molecular weight molecules such as *N*-acylhomoserine lactone (AHL) [4], furanosyl borate diester (Autoinducer-2) (AI-2) [5], 4,5-dihydroxy-2,3-pentanedione (DPD) [6], 3-hydroxypalmitic acid methyl ester (3OH-PAME) [7], *cis*-11-methyl-2-dodecenoic acid (diffusible signal factor) (DSF) [8], 2-isocapryloyl-3R-hydroxymethyl-γ-butyrolactone (A-factor) [9], diketopiperazines (DKP) [10], 2-heptyl 3-hydroxy-4-quinolone (*Pseudomonas* quinolone signal) (PQS) [11] and 4-hydroxy-2-heptyl-quinoline (HHQ) [12] to high molecular weight molecules such as oligopeptide autoinducer [13].

Arguably, the well understood QS mechanism mediated by AHLs as signalling molecules is used by most of the Gram-negative bacteria. This mechanism involves synthesis of AHLs (by LuxI, AHL synthase), channeling of AHLs (notably the long-chain AHLs), binding to the cognate receptor (LuxR protein) and activation of QS-mediated genes [14,15]. Myriad structural variants of the basic AHL molecules have been discovered and they vary in length and degree of saturation of the acyl side chain as well as in the functional group located at C3 [16]. Over 100 species of Gram-negative proteobacteria are known to produce AHLs, regulating the expression of diverse physiological activities: bioluminescence, biofilm formation, synthesis of antibiotics, synthesis of exoenzymes and nodulation [3]. AHL production has also been observed in extremophiles such as the haloalkaliphilic archaeon *Natronococcus occultus* [17] which lives in an alkaline biotope (pH10) and the acidophilic gamma-proteobacterium *Acidithiobacillus ferrooxidans* [18–20]. In 2008, *Gloeothece* sp. strain PCC6909, a cyanobacterium, was found to produce AHLs or AHL-like molecules [21]. More importantly, QS regulates virulence determinants in several Gram-negative pathogenic bacteria belonging to both aquaculture (*Vibrio anguillarum*, *Aeromonas salmonicida*) and plant-associated genera (*Erwinia caratovora*, *Agrobacterium tumefaciens*) (for reviews, see [3,15]), and human pathogens (*Pseudomonas aeruginosa, Burkholderia cepacia* and *Yersinia pseudotuberculosis*).

Interference of AHL-dependent QS, or commonly known as quorum quenching (QQ), has been regarded as the novel way to control bacterial infections. QQ can be achieved in several ways. First, inhibition of AHL biosynthesis can be achieved by inhibiting the enzymes involved in the biosynthesis of acyl chain (acyl-acyl carrier protein) (ACP) and *S*-adenosylmethionine synthase [22], as well as the LuxI homolog proteins. Second, destruction of the QS signalling molecules will prevent them from accumulating. Two major enzymes that degrade AHL have been discovered, *i.e.*, AHL lactonase and AHL acylase. Dong *et al.* proposed that inhibition of the AHL efflux protein can be one of the mechanisms in interfering QS as this will cause failure in accumulation of AHLs in the environment [14]. Third, inhibition of LuxR homolog proteins (QS receptors) is able to attenuate QS-dependant virulence [23,24]. For example, halogenated furanones produced by *Delisea pulchra* inhibit AHL-dependent gene expression by displacing the AHL signal from its reporter protein [25]. Several comprehensive reviews on QS analogues that antagonize QS are available [26–28].

## 2. Quorum Quenching Enzymes

**Figure 1.**
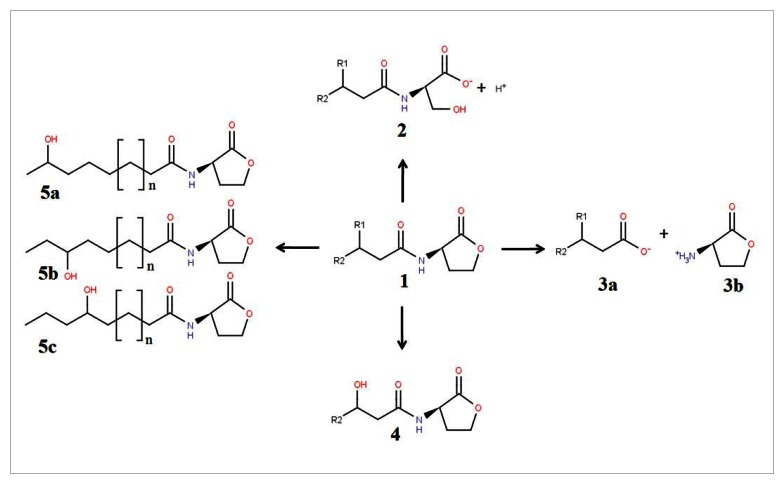
The signalling molecule *N*-acyl homoserine lactone (AHL) (**1**) can be degraded by lactonase, yielding *N*-acyl homoserine (**2**). Acylase cleaves the amide bond of AHL by releasing fatty acid (**3a**) and homoserine lactone (**3b**). Oxidoreductases from *Rhodococcus erythropolis* W2 and *Burkholderia* sp. strain GG4 inactivate AHL by substituting the oxo group at the C3 with hydroxy group (**4**). Similarly, CYP102A1 from *Bacillus megaterium* reduces the acyl chain of AHL at the ω-1, ω-2 and ω-3 positions (**5a**, **5b**, **5c**). R1 corresponds to the side chain of the C3 position (3-oxo-, 3-hydroxy- and 3-unsubstituted). R2 corresponds to the acyl side chain of AHL and n corresponds to the alkyl group.

According to Dong and Zhang, the chemical structure of AHLs suggests that four different ways of degradation may occur mediated by lactonase, decarboxylase, acylase and deaminase [29]. Of these, only two types of QQ enzymes have been found, namely lactonase and acylase. The former hydrolyzes the lactone and the latter acyl chains (Figure 1). An AHL lactonase hydrolyzes the ester bond of the lactone ring, forming acyl homoserine, which renders the signalling molecules incapable of binding to their target transcriptional regulators, thus attenuating the QS mechanism [30,31]. An AHL acylase, also known as AHL amidohydrolase, cleaves the peptide (amide) bond of the lactone ring to release a fatty acid and homoserine lactone, causing significant reduced function of the signalling molecule [32].

### 2.1. Lactonases

The first QQ enzyme, AiiA, was purified from the Gram-positive *Bacillus* sp. strain 240B1 [31], and was later characterized as an AHL hydrolase [30]. The 250-residue-long amino acid sequence shows a conserved sequence motif of ^104^HXHXDH^109^∼H^169^, similar to the Zn^2+^ binding motif of several metallohydrolases which belong to the metallo-β-lactamase (MBL) superfamily of protein [31]. This superfamily consists of a great diversity of protein set, ranging from no metal, mononuclear zinc, dinuclear zinc to dinuclear iron active sites. At least 17 different catalytic activities and mechanisms have been reported within this superfamily, including nitric oxide and oxygen reduction [33]. The crystal structure of AiiA from *Bacillus thuringiensis* strain BTK shows the presence of two Zn^2+^ ions at the active centre of the enzyme and the presence of the HXHXDH∼H serves as the signature motif for the enzyme to be classified as AHL lactonase [34]. The initial report of Wang *et al.* [35] suggested that AHL lactonases were not metal-dependent; however, subsequent work on two different isoforms, *i.e.*, AiiA lactonases from *B. thuringiensis* strain BTK [34,36] and *B. thuringiensis* strain BGSC 4A3 [37], has shown that both are dinuclear metalloproteins with two Zn^2+^ ions bound in the proximity of each other at the active site. Interestingly, these metal ions are essential for the cleavage of the ester bond on the lactone ring and the proper folding of the enzyme [38]. Besides the zinc ions, the highly conserved metal-binding histidine or the aspartate residues and Tyr-194 in the AiiA demonstrate their requirement for catalytic activity [34,39]. The diversity and polymorphism of AiiA, mechanism and crystal structure analysis of the AiiA lactonase from *B. thuringiensis* have been discussed in detail [38,40,41].

Homologs of AiiA have been discovered in many bacteria belonging to the *Bacillus* genus [42]. Interestingly, members from the Bacillaceae, *i.e.*, *Geobacillus kaustophilus* HTA426 [43] and *Geobacillus caldoxylosilyticus* YS-8 [44], have been found to produce thermostable lactonase. According to Chow *et al.* [43], the thermostable lactonase from *G. kaustophilus* strain HTA426, namely GKL, does not belong to the MBL superfamily but to the phosphotriesterase (PTE)-like lactonase (PLL) group of enzymes within the amidohydrolase superfamily. GKL exhibits a low metal-dependent AHL lactonase activity while Zn^2+^-reconstituted GKL displays a substrate preference for medium to long-chain AHLs (≥8 carbons) as well as substrates like γ-nonalactone and δ-nonalactone. Furthermore, GKL exhibits a relatively lower paraoxonase activity, indicating that, like other members of the PLLs, the native substrate profile of GKL does not involve phosphate ester [45]. Similarly, a highly thermostable phosphotriesterase-like lactonase produced by *G. stearothermophilus* has a relatively low catalytic efficiency against the tested AHL, *i.e.*, *N*-hexanoyl-homoserine lactone [46].

*Mycobacterium avium* subsp. *paratuberculosis* K-10 has been reported to produce a PLL named MCP. This lactonase is a member of the amidohydrolase superfamily and it shares sequences identities of 92 and 59% with PPH (lactonase from *M. tuberculosis*) and AhlA (lactonase from *R. erythropolis*), respectively [45]. According to Chow *et al.* [47], MCP hydrolyzes medium to long-chain AHLs, *i.e.*, C7-HSL, C8-HSL, 3-oxo-C8-HSL, C10-HSL and C12-HSL.

In the past few years, besides *Bacillus*, several lactonase-producing bacteria along with their lactonase have been identified, *i.e.*, AttM and AiiB of *A. tumefaciens* [48,49], AhlD of *Arthrobacter* [50], AhlK of *Klebsiella pneumonia* [50], AidH of *Ochrobactrum* [51], AiiM of *Microbacterium testaceum* [52], AhlS of *Solibacillus silvestris* [53] and QsdA of *Rhodococcus* strains W2, LS31 and PI33 [54,55] (Table 1). Several bacteria have been identified to be lactonase- or possibly lactonase-producing bacteria although no further work has been done on the QQ mechanism. They are *Acinetobacter* [56,57], *Nocardioides kongjuensis* [58], *Chryseobacterium* [59], *Sphingopyxis witflariensis* and *Bosea thiooxidans* [60]. We have also identified from staphylococci lactonase activity which efficiently degrades a wide range of AHLs (unpublished data).

Besides members from the Eubacteria domain, an archaeon, *Sulfolobus solfataricus* strain MT4, has been discovered to produce a QQ enzyme [61] known as SsoPox, which is an aryldialkylphosphatase (EC 3.1.8.1), a member of the PLL group of the amidohydrolase superfamily of metalloenzymes [45]. It possesses a (β/α)_8_ barrel fold containing a binuclear divalent metal center composed of Co^2+^ and Fe^3+^ that assists with substrate binding and an activated water molecule which is involved in the hydrolysis reaction [62]. This enzyme was first reported to hydrolyze organophosphate but subsequent studies suggested that it hydrolyzes AHLs (with acyl chain length >C8). Interestingly, this enzyme shares no sequence similarities with AiiA lactonase [81].

Attempts have been made to isolate lactonases QlcA, BpiB01, BpiB04, BpiB05 and BpiB07 from unculturable bacteria through metagenomic approaches (Table 1). The majority of these lactonases are from unique protein families such as BpiB04 which is a member of glycosyl hydrolase family [64] and BpiB05 which is a member of dienelactone hydrolase family [65], in contrast to the other lactonases, which are normally members of the metallo-β-lactamase superfamily and PTE superfamily, from culturalble bacteria. QQ enzymes are therefore more diverse than currently thought. Novel growth media for enrichment of unculturable bacteria and metagenomic approaches are desirable methods to study novel QQ enzymes and the QQ mechanisms [82].

### 2.2. Acylases

An AHL acylase, which degrades the amide bond of a wide variety of AHL molecules yielding homoserine lactones and fatty acids that serve as the sole energy and nitrogen sources, was isolated from *Variovorax paradoxus* VAI-C [77], but the gene encoding the enzyme has not been identified. Hence, enzymology study of this AHL acylase is lacking though *V. paradoxus* has been shown to degrade a wide range of organic substrates including aromatic compounds [83–87].

The second reported AHL acylase is the AiiD from *Ralstonia* sp. XJ12B, which degrades and grows equally rapidly with short- and long-chain AHLs. AiiD, a 794-amino-acid polypeptide, shares significant similarities with aculeacin A acylase (Aac) from *Actinoplanes utahensis* and cephalosporin acylase from *Brevundimonas diminuta* [32]. This enzyme belongs to the N-terminal nucleophile aminohydrolase (Ntn hydrolases) superfamily. AiiD undergoes post-translational modification (autoproteolytic processing and catalysis) to form a functional enzyme [32]. Chen *et al.* reported a 795-amino-acid acylase, Aac, from *Ralstonia solanacearum* GM1000, a phytopathogen, and it has 83% similarity with AiiD of *Ralstonia* sp. XJ12B [72]. AaC is active against AHLs with acyl side chains > C6 regardless of the substituent group at the C3 and the AHL molecules.

Shortly after the discovery of AiiD, another AHL acylase, namely PvdQ (PA2385), from *P. aeruginosa* was reported by Huang *et al.* [68]. It has a relatively high homology with AiiD from *Ralstonia* sp. *P. aeruginosa* is a 3-oxo-C12-HSL producing bacterium. Thus, PvdQ might be involved in the regulation of the self-produced 3-oxo-C12-HSL. The post-translational processing of the acylase PvdQ, *i.e.*, autoproteolytically activated excision of a 23-residue prosegment, and the hydrolysis reaction type are similar to those of the beta-lactam acylases. These findings suggest that PvdQ is a member of the Ntn hydrolase superfamily and comprises an α/β heterodimeric Ntn hydrolase fold, bearing an α-subunit and a β-subunit of approximately 18 and 60 kDa, respectively [88]. The open reading frame (ORF) of *pvdQ* encodes a 726-amino-acid polypeptide with a theoretical molecular mass of 84.0 kDa [69]. PvdQ degrades only long-chain AHLs. It shows a broad-range AHL degrading activity, because the substituents at C3 of the AHL do not affect its activity. Lin *et al.* showed that *pvdQ* knockout mutants are able to metabolize 3-oxo-C12-HSL as the sole energy source, indicating the presence of another enzyme(s) that contribute to the degradation of AHLs [32,68]. The detailed description of the structure of PvdQ and the mechanism of AHL hydrolysis by PvdQ are discussed in Bokhove *et al.* [88].

Another AHL acylase, called QuiP (PA1032), from *P. aeruginosa* was discovered in 2006. QuiP is one of four Ntn hydrolase homologs encoded by the *P. aeruginosa* genome [70]. QuiP shares 21 and 23% amino acid identities with PvdQ and AiiD, respectively. QuiP has a very high degree of similarity to the Ntn hydrolase family of proteins and it is predicted to be cleaved into four peptides: a signal sequence, two peptides corresponding to the α and β subunits of the natural enzyme, and a spacer protein [70]. QuiP has preference to degrade long-chain AHLs (with the acyl side chain longer than six carbons) and it does not degrade AHLs with short acyl side chains. QuiP is constitutively expressed during growth and its activity is sufficient to degrade long-chain AHLs produced by the host. The expressions of QuiP, PvdQ and AHL synthases are believed to regulate the production of AHL molecules in order for the bacteria to communicate and to ensure that cell-to-cell communication is not disrupted. There is a remote possibility that *quiP* is expressed when the bacteria are in the biofilm state [70].

In 2009, Shepherd and Lindow [71] reported that *Pseudomonas syringae* strain B728a produces two acylases, HacA (Psyr_1971) and HacB (Psyr_4858), which belong to the Ntn hydrolase superfamily. The former is a secreted acylase which degrades only long chain-AHLs (AHLs with eight carbons or more), while the latter is a non-secreted acylase which degrades a wide range of AHLs (from C6-HSL to C12-HSL). HacA and HacB share 55 and 68% identities with PvdQ and PA0305, an uncharacterized protein from *P. aeruginosa*, respectively [71].

An acylase-encoding gene, *ahlM*, has been discovered in *Streptomyces* sp. strain M664 [75]. AhlM, a heterodimeric protein with subunits of approximately 60 and 23 kDa, is a member of the Ntn hydrolase family. Post translational processing cleaves the first 35 amino acids at the amino-terminal of the α subunit of the protein during maturation. AhlM is more active against unsubstituted rather than 3-oxo-substituted long-chain AHLs. It degrades C8-HSL, C10-HSL and 3-oxo-C12-HSL. In addition, AhlM hydrolyzes penicillin G and releases 6-aminopenicillanic acid, indicating that it is an enzyme with broad substrate specificity [75].

Acylase-type AHL degradation activity has been discovered in *Anabaena* sp. PCC7120, a nitrogen-fixing cyanobacterium. This acylase named AiiC and encoded by the gene *all3924*, shows homology to QuiP of *P. aeruginosa* PAO1. The crude cell extract of *Anabaena* degrades a broad range of AHLs, though it prefers the long-chain AHLs, regardless of the substitution group at the C3 position. AiiC might be important in controlling nitrogen fixation, as there is a putative binding site for the NtcA in the *aiiC* promoter [66].

Other bacteria that produce acylase or acylase-like enzyme include *Shewanella* [74], *Tenacibaculum maritimum* [76], *R. erythropolis* W2 [73], *Comamonas* sp. strain D1 and *Comamonas testosterone* [67,89]. According to Uroz *et al.*, *Comamonas* strain D1 is able to degrade a broad range of AHLs ranging from 4 to 16 carbons, with or without 3-oxo or 3-hydroxy substitutions at C3 [67].

In 2003, Xu *et al.* reported that the commercial porcine kidney acylase I (EC 3.5.14) is able to deacylate C4-HSL and C8-HSL to produce l-homoserine [90]. According to Xu *et al.*, the optimal pH for this enzyme is 10 at 23 °C and the optimal temperature of 76 °C at pH 9. A nanofiltration membrane immobilized with this enzyme has shown great anti-biofouling feature by suppressing extracellular polymeric substances secretion and thus biofilm maturation [91,92].

Table 2 summarizes the various roles of QQ enzymes.

### 2.3. Signalling Confusion

Recently, another group of AHL inactivation enzymes has been reported and it includes oxidoreductases which modify but not destroy AHLs. Uroz *et al.* have shown that the AHL oxidoreductase from *R. erythropolis* strain W2 reduces 3-oxo-substituted AHLs to their corresponding 3-hydroxy derivatives [73]. This oxidoreductase shows a high affinity for long-chain 3-oxo-AHLs (at least eight carbons). Its oxidoreductase activity is not stereospecific as it reduces both d- and l-isomers of 3-oxo-C12-HSL. Furthermore, this enzyme is not specific solely to AHLs with a 3-oxo-substituent. It is able to reduce AHL derivatives such as *N*-(3-oxo-6-phenylhexanoyl) homoserine lactone, which contains an aromatic acyl chain substituent, and 3-oxododecanamide, which lacks the homoserine lactone ring [73].

The modified AHL, though still functionally active, may fail to bind specifically to its LuxR receptor, thus causing disturbance to the activation of QS-mediated genes regulated by a particular AHL. In this paper, we call this phenomenon “signalling confusion” which does not involve destruction of the AHL structures like the lactone ring or the *N*-acyl side chain. This phenomenon can be illustrated by the oxidoreductase activity of *Burkholderia* sp. GG4, which specifically reduces the oxo group at the C3 position of the oxo-AHLs into the corresponding hydroxy-AHLs [57]. Unlike *R. erythropolis* strain W2, which first modifies AHLs signal molecules through an oxidoreductase activity and then degrades the modified AHLs through an amidolytic activity [73], *Burkholderia* sp. GG4 does not degrade the modified AHL, namely hydroxy-AHL. While other bacteria may still use the modified AHL as QS molecules, the producer of this oxo-AHL will be deprived of its cognate QS oxo-AHL to bind to its LuxR receptor. As shown by Chan *et al.* [57], even though *Erwinia carotovora* produces 3-oxo-C6-HSL, co-culture of *Burkholderia* sp. strain GG4 and *E. carotovora* attenuates maceration of the potato tuber. This is because the signalling molecule 3-oxo-C6-HSL is modified (but not destroyed) by the oxidoreductase of *Burkholderia* sp. GG4 [57]. The resulting hydroxy-AHL may disturb QS bacteria that rely on it as QS molecules through pre-mature triggering of target genes via the binding of this non-native hydroxy-AHL to its receptor. This “signalling confusion” will have impact on QS in microbial microhabitats, especially those that rely on oxo-AHLs and, to a certain extent, hydroxy-AHLs as signalling molecules.

Interestingly, CYP102A1, a widely studied cytochrome P450 from *B. megaterium*, can oxidize AHLs and the lactonase- and acylase-degraded products of AHLs, *i.e.*, acyl homoserines and fatty acids, at the ω-1, ω-2 and ω-3 positions (Figure 1) [78]. The action of this oxidizing enzyme is independent of the presence or absence of the 3-oxo-group at C3 and occurs whether the lactone form is oxidized or the hydroxy-acid form is oxidized and then recyclized.

Thus, oxidation of AHLs yields hydroxy-AHLs. CYP102A1 exhibits a lower preference for modification of the l-isomers than the racemic mixture, but both stereoisomers bind better than the comparable fatty acid. The *A. tumefaciens* NTL4 bioassay shows that oxidation of the AHLs decreases their QS activity, but not as much as lactonolysis [78].

Recently, a shot-chain dehydrogenase/reductase (SDR) which reduces 3-oxo-C12-HSL to 3-hydroxy-C12-HSL has been discovered via the metagenomic approach. This small polypeptide (30 kDa) is a new member of the SDR superfamily which requires NADPH as a cofactor. BpiB09 is the first NADP-dependent SDR derived from a soil metagenome [79]. It has a typical Rossmann fold with a central beta sheet flanked by three helices at both sides. The active substrate binding site is located in the variable C-terminal region by the presence of a catalytic tetrad. The gene that encodes this NADP-dependent oxidoreductase shares 57% nucleotide identity with a possible SDR from *Acidobacterium capsulatum*. Expressing *bpiB09* in *P. aeruginosa* PAO1 leads to decreased motility and reduced pyocyanin and 3-oxo-C12-HSL production, while it attenuates biofilm formation and virulence on *Caenorhabditis elegans*. It also leads to significant down-regulation of at least 38 QS-dependent genes in *P. aeruginosa* PAO1. According to Bijtenhoorn *et al.*, BpiB09 reduces 3-oxo-C12-HSL to 3-hydroxy-C12-HSL, causing the failure of LuxR homolog protein to recognize the signalling molecule. Furthermore, BpiB09 interferes with the synthesis of 3-oxo-C12-HSL by reducing free 3-oxo-acyl-ACP in the cell [79].

### 2.4. Paraoxonases

Paraoxonases (PONs), including PON1, PON2 and PON3, are mammalian enzymes that catalyze the hydrolysis and inactivation of various compounds such as organophosphates, esters and lactones [102,103]. PONs are calcium-dependent enzymes whose activities could be inhibited by EDTA, a metal-chelating agent. This can be rescued by supplementation of Ca^2+^. In human, the genes that encode these three enzymes are located adjacent to each other on the long arm of chromosome 7q21.3-22.1 [104]. These three enzymes exhibit a high level of similarity in their structural features, having about 65% identity at the amino acid level [104]. In 2007, Harel *et al.* showed that PON1 is a 6-bladded β-propeller with a unique active-site lid and two Ca^2+^ ions in its central tunnel [105]. PON1 can hydrolyze a wide range of compounds [102] and is capable of reversing the hydrolysis reaction, *i.e.*, lactonization, of γ- and δ-hydroxycarboxylic acids [106]. The detailed catalytic mechanism of PON1 is discussed in Harel *et al.* [105].

PONs play a key role in organophosphate detoxification, lipid metabolism, and prevention of atherosclerosis. They are believed to play an important role in defense against bacterial infections as they are able to degrade AHLs, inactivating QS among nosocomial bacteria [102,106,107]. All three PONs hydrolyze 3-oxo-C12-HSL produced by *P. aeruginosa* [108]. Stoltz *et al.* have demonstrated that PON2-deficiency enhances *P. aeruginosa* QS in murine tracheal epithelia, suggesting that PON2 plays a pivotal role in protecting the host from bacterial infection [109]. It modulates oxidative stress by attenuating the production of reactive oxygen species (ROS) by *P. aeruginosa* virulence factor pyocyanin. Phylogenetic analysis shows that PON2 is the oldest member of the family from which PON3 and next PON1 arose [102,110]. PON3 evolved an active site larger than that of PON2, capable of accommodating larger substrates such as satin lactones and spironolactone. On the other hand, PON1 evolved a smaller active site than that of PON2, allowing it to hydrolyze non-substituted, short-chain-substituted lactones [102,110].

PONs appear to be important in modulating oxidative stress and protecting the cardiovascular systems against diseases that result from deficiencies in modulating oxidative stress [111]. Horke *et al.* reported a mechanism by which bacteria may subvert the protection provided by PON2 [112]. According to them, 3-oxo-C12-HSL and some virulence factors of *P. aeruginosa*, such as pyocyanin and flagellin, induce cytosolic Ca^2+^ influx, causing down-regulation of PON2 mRNA transcription, which results in the reduction of PON2 protein translation and PON2 hydrolytic activity [112].

## 3. Why Quorum Quenching

Soon after the discovery of lactonase (AiiA) produced by soil bacilli [31], other QQ enzymes from a wide variety of bacteria have been confirmed, namely AttM (*A. tumefaciens*), AhlD (*Arthrobacter* sp.), QsdA (*R. erythropolis*), PvdQ and QuiP (*P. aeruginosa*). QQ bacteria can be divided into three phyla, *i.e.*, Firmicutes (*Bacillus* sp., *Geobacillus* sp. and *S. silvestris*), Actinobacteria (*Arthrobacter*, *M. testaceum*, *R. erythropolis*, *M. avium* and *Streptomyces*) and Proteobacteria (*Agrobacterium*, *V. paradoxus*, *R. solanacearum*, *Shewanella* sp., *P. aeruginosa*, *Comamonas* sp., *Burkholderia* sp., *Acinetobacter* sp. and *Delftia* sp.). Interestingly, *Anabaena* sp., a member of cyanobacteria, has been discovered to exhibit lactonase activity. Similarly, *Tenacibaculum maritimum*, a member of Bacteroidetes, produces acylase. Several recent papers reported the discovery of novel QQ enzymes via the metagenomic approach (Table 1). All these show that the genes encoding QQ enzymes are widely conserved among many prokaryotic microorganisms [29].

QQ enzymes have been thought to solely play an important role in interfering QS. The well studied AiiA lactonases of *B. thuringiensis* has been shown to quench the virulence of the phytopathogen *E. carotovora* by inactivating its AHL signals [113]. Czajkowski and Jafra have reported that bacteria produce QQ enzymes in order to ensure success in competition for the limited natural resources [114]. Park *et al.* revealed that the AiiA lactonase of *B. thuringiensis* plays an important role in rhizosphere competence of *B. thuringiensis* [96]. The *aiiA*-defective mutant has a relatively lower survival rate, competency and adaptability compared to the wild type [96]. These findings collectively suggest that the AiiA lactonase plays an important role in microbial competition. However, Kaufmann *et al.* proposed that the lactonase of *Bacillus* sp. plays a crucial role in controlling the toxicity effect of AHLs and prevents the formation of tetramic acid derivatives, *i.e.*, nonenzymatical products of Claisen-like condensation reaction of the 3-oxo-AHLs [97]. Tetramic acid derivatives are bactericidal agents which act against Gram-positive bacteria. Furthermore, tetramic acid derivatives are able to chelate diverse metal cations, such as iron, forming metal complexes believed to act as primordial siderophore. Thus, by degrading 3-oxo-AHLs, the toxicity of AHLs is abated, formation of tetramic acid derivatives prevented, competition for iron in the natural environment decreased, signalling pathway of competitors interfered and bacterial survival in the natural environment enhanced [97]. An interesting opinion that contradicts this suggestion has been posted by Zhou *et al.*, who suggested that *Bacillus* species, especially *B. cereus*, have different needs in terms of the ecological environment and nutritional sources compared to those of Gram-negative bacteria, e.g., *E. carotovora. B. cereus* has a preference for protein and amino acid substrates. On the other hand, *E. carotovora* prefers nutrients derived from plants. Therefore, it is unlikely there is competition between these two bacteria [115,116].

Destruction of the AHL structure is not the only means for *Bacillus* to exert its QQ effect. It has oxidoreductase capable of inactivating the AHL molecule by oxidizing the acyl chain at the ω-1, ω-2, and ω-3 carbons [78]. This mechanism of QQ decreases the QS activity but not as much as lactonolysis which inhibits QS completely. It is hypothesized that this oxidoreductase makes acyl homoserine more membrane permeable, thus preventing the accumulation of degraded AHL products inside the cell. Furthermore, it makes acyl homoserine and AHLs more water soluble, thereby enhancing their diffusion out of the cell. As 3-oxo-AHLs are converted into tetramic acid derivatives nonenzymatically, oxidizing the acyl chain helps to detoxify the AHLs before the conversion takes place. The oxidization of the acyl chain might be the first of AHL metabolic pathway [78].

*V. paradoxus* is first bacterium shown to metabolize different AHLs as the sole carbon, nitrogen, and energy sources [77]. Later, Park *et al.* [50] reported a Gram-positive bacterium, *Arthrobacter* sp. ISN110, that produces AhlD lactonase and can degrade and use various AHLs for energy and growth. Yoon *et al.* [58] also reported that another Gram-positive bacterium, *N. kongjuensis*, is able to grow on C6-HSL and use the AHL degradation products as the carbon source [58].

The phytopathogen *A. tumefaciens* expresses two lactonases, *i.e.*, AttM and AiiB, which serve as the modulators of QS-regulated conjugation and transfer of tumour inducing (Ti) plasmid [48,49] by regulating the level of 3-oxo-C8-HSL. Expression of *aiiB* is induced by plant signals such as opines, agrocinopines A and B. On the other hand, expression of *attM*, which is part of the *attKLM* operon, is induced by succinic semialdehyde, γ-hydroxybutyrate, γ-butyrolactone (GBL), salicylic acid and γ-aminobutyrate [93, 117]. AiiB modulates the conjugation frequency of the Ti plasmid and the emergence of tumour. AttM lactonase contributes to the fitness of *A. tumefaciens* in the plant tumour. Both AiiB and AttM modulate the level of 3-oxo-C8-HSL. The QS pathways and the QQ enzymes of *A. tumefaciens* combine to contribute to optimal expression of virulence functions in *A. tumefaciens* [49]. Thus, AttM and AiiB play an important role in the regulatory machinery of QS in *A. tumefaciens*. However, this view has been challenged by Khan and Farrand who showed that AttM lactonase (or BlcC for γ-butyrolactone catabolism by Khan and Farrand) does not degrade AHL bound to TraR and the overexpression or null mutation of *blcC* does not significantly affect the conjugation competence and transfer of Ti plasmid [94]. Besides, the *blc* operon is not widely distributed in the genus of *Agrobacterium. Agrobacterium* spp. that harbour *blcC* exhibit a significantly better growth on minimal medium with GBL as the sole source of carbon. Thus, the function of the *blc* operon concerns the catabolism of butyryl compounds rather than the QS-regulated Ti plasmind conjugative transfer. BlcC degrades GBL to a product which may be eventually converted to succinic acid, an intermediate in the citric acid cycle [94].

*P. aeruginosa* has been shown by Huang *et al.* [68,70] to produce two acylases, PvdQ and QuiP. They suggested that QuiP may play a role to distinguish subpopulation spatially, especially in the biofilm state [70]. The gene that encodes PvdQ acylase is located in the *pvd* locus, essential for the regulation of pyoverdine biosynthesis [98]. PvdQ might play a role in the utilization of AHL, maturation of pyoverdine siderophore and regulation of 3-oxo-C12-HSL-regulated physiological functions [68]. The last possible role is supported by Sio *et al.* [69] who provided experimental evidence that PvdQ modulates QS-regulated virulence phenotypes. Under iron-limiting conditions, deletion of *pvdQ* led to attenuation of virulence, decreased swarming motility and failure to form biofilm. Hence, PvdQ might be involved in the biosynthesis of pyoverdine and regulation of iron homeostasis. Its role in iron sequestration precedes its acylase activity under iron-limiting conditions [99].

According to Wang *et al.* [100] and Yeung *et al.* [118], *lasI* mutant and *pvdQ* mutant exhibit reduced swarming motility, suggesting that a specific concentration of 3-oxo-C12-HSL is crucial for the swarming motility. The degraded AHL products of PvdQ may serve as a signal during swarmer cell differentiation [118]. In 2011, Wang *et al.* [100] made an interesting proposal that as both biofilm formation and swarming motility are dependent on a functional flagellum, PvdQ may play a role in regulating the flagellum-dependent motions. This in turn facilitates the decision-making mechanism between biofilm formation and swarming motility [100]. Wang *et al.* also suggested that PvdQ might play an important role in antibiotic resistance by altering the membrane permeability [100]. PvdQ may change the outer membrane permeability by up-regulating the lipopolysaccharide-related operon [100], and alter the expression of the outer membrane TonB-dependent receptors, OprF (which is involved in cell adhesion, binding of gamma interferon and activation of QS-pathway) [119,120] and OprD (which facilitates the transport of basic amino acids and imipenem) [121]. In a recent study, Hannauer *et al.* demonstrated that *pvdQ* mutant produces pyoverdine I precursors with a myristic or a myristoleic acid chain and an unformed chromophore [101]. This leads to the suggestion that PvdQ plays a role in removing the acylated fatty acid chain or the non-fluorescent precursor prior to the cyclization of chromophore [101]. These acylases might be important in preventing premature production of virulence factors that could trigger the immune response of the host. In summary, PvdQ might play a role in the utilization of AHL, regulation of QS-dependent phenotypes, biosynthesis of pyoverdine and elevation of antibiotic resistance.

Like *P. aeruginosa* and *A. tumefaciens*, *R. solanacearum* exhibits both QS and QQ systems. It possesses *aac* gene which encodes acylase that degrades long chain AHLs. According to Chen *et al.*, *R. solanacearum* metabolizes AHL degradation product, *i.e.*, fatty acids, by β-oxidation during cultivation [72]. Thus, acylase may be involved in the metabolism of AHLs. This enzyme may modulate QS pathways by using a unique signal turnover mechanism [32,72]. Lin *et al.* proposed that the acylase of *Ralstonia* plays an important role during oligotrophic nutrient scavenging from the natural environment [32].

Interestingly, *R. erythropolis* possesses three different types of QQ enzymes, namely oxidoreductase, acylase and lactonase [55,73]. However, the roles of all these QQ enzymes remain unclear. Several neighboring sequences of the lactonase-encoding gene play an important role in the metabolism of fatty acids, such as acyl coenzyme A synthase and FadR peptide analogous to a fatty acid biosynthesis regulatory protein. Thus, the lactonase produced by *R. erythropolis* might be involved in fatty acid metabolism [55].

*Chryseobacterium* is a member of the Cytophaga-Flavobacterium-Bacteroides (CFB) group whose lactonase degrades AHL. It uses the degraded product for growth and energy, and might play a role in providing protection to plants from pathogens [59]. Hence, *Chryseobacterium* and its plant host live in symbiosis. Similarly, the endophytic Gram-positive bacterium *M. testaceum* that produces AiiM lactonase may play a role in interfering with the QS pathways of pathogens, thereby providing protection to the host against pathogens invasion [52]. AiiC acylase produced by *Anabaena*, a member of cyanobacteria, is believed to play a role in interfering with the communication system within the complex microbial communities [66]. AiiC might be important in controlling the exogenous AHLs, but the rationale behind it remains unknown. AHLs have been shown to inhibit nitrogen fixation pathway in *Anabaena*. In addition, with the presence of nitrogen source from the natural environment, 3-oxo-C10-HSL exhibits cytotoxic effect on *Anabaena* [95]. Thus, the presence of QQ enzyme might have an important role in defense mechanism.

In summary, several roles of QQ enzymes have been postulated, from interference of QS to metabolism of AHL as the source of carbon and nitrogen, detoxification, regulation of physiological functions, and symbiotic interaction with the host. To date, there is no experiment showing a conclusive link between QQ and the ability to gain a competitive advantage [122]. There are still a number of bacteria in which the roles of their QQ enzymes remain enigmatic. Hence, further investigation is needed to gain insight into the role of QQ enzymes.

## 4. Making Use of Quorum Quenching: From Interference with Bacterial Communication to Application

### 4.1. Quorum Quenching in Pharmacology

Bacteria use QS to regulate the production of virulence factors [123] and interestingly, this type of communication mechanism is not limited to same species communication. Communication between bacterial species has been reported, especially in a polymicrobial biofilm [124]. Besides synchronizing the colonization and invasion processes, QS has been shown to downregulate the host's immune system by suppressing the production of tumor necrosis factor-alpha (TNF-α) and interleukin-2 of macrophages [125]. All these findings together with the emergence of antibiotic resistant bacteria and shortage of new antibiotics in treating infectious diseases make QS an interesting target to counter infections.

Since the first report on the interference of QS by secondary metabolites of the macroalga *D. pulchra*, efforts to search for QS-interfering compounds have been intensified [126]. Compounds which are able to antagonize QS by disrupting the synthesis of signalling molecule, inhibiting the diffusion of signalling molecules, blocking the signalling molecules from binding to the corresponding receptor protein, or preventing the signal transduction upon binding of signalling molecules to the receptors, with the condition not creating selective pressure, could therefore be the potential anti-virulence drugs. In principle, QQ enzymes, such as lactonases, acylases and oxidoreductases, are suitable candidates as anti-virulence drugs too, because they inactivate signalling molecules without interfering with the enzymatic mechanism inside the bacterial cell, thereby reducing the selective pressure.

Other than regulating the invasion capability of bacteria, QS has been reported to play an important role in the development of biofilm notably in the lungs of cystic fibrosis (CF) patients. Biofilm contributes to the rate of morbidity and mortality [127], health cost, device-associated infections and catheter-related bloodstream infections [128,129]. As biofilm is QS-regulated, thus, by disrupting QS, the formation of biofilm could be prevented. Interestingly, in most cases, interference of QS does not prevent the formation of biofilm, but it makes the biofilm more susceptible to antimicrobial compounds and immune responses of the host [130]. Estrela and Abraham recommended a new approach in controlling biofilm and biofilm-associated infections through a combination of biofilm-destroying compounds and antibiotics [130]. QQ compounds could work synergistically with antibiotics for enhancement of treating QS-dependent infections.

However, before QQ compounds and enzymes are used, due consideration must be given to their toxicity, stability, delivery, reaction with the gastric fluids (if administered orally), source and cost of synthesis. The last two factors are related to the continuous supply of QQ compounds and enzymes. Although QQ may represent a promising strategy, there are several drawbacks. For instance, not all pathogenic bacteria use QS to control virulence determinants. Furthermore, resistance towards QQ, increased virulence owing to the dispersal of biofilm and the chances of triggering hypersensitivity reaction in patients may occur.

### 4.2. Quorum Quenching and Anti-Biofouling

Biofouling is the undesirable accumulation of microorganisms, plants, algae and invertebrate animals on structures immersed in the marine environment, and it has a severe impact on the shipping industry, fishing and aquaculture industry and oceanographic sensor [131]. The major cost associated with biofouling is contributed by the increase of fuel consumption due to the increased frictional drag [132]. The estimated overall cost associated with hull fouling for the US Navy's current hull husbandry practices is approximately US$56 million per year [133].

In 2008, the International Maritime Organization and Marine Environmental Protection Committee banned the use of toxic organotin tributyltin-based paint product as anti-biofouling agents. Since then the search for environment friendly anti-biofouling or biofouling-release agents has intensified [134]. One of the most recommended approaches in inhibition of biofouling is via anti-biofouling compounds, in which some QQ compounds such as betonicine and furanone may be useful [135]. QQ compounds inhibit the QS mechanism of bacteria, thereby causing low bacterial attachment or recruitment and inhibiting the formation of biofilm [136].

Besides using QQ compounds as the anti-biofouling approach, material scientists have synthesized anti-biofouling surface topographies fabricated in nontoxic poly(dimethyl siloxane) elastomers to prevent biofouling [137]. However, the major drawbacks of this technology are its low durability, susceptibility to slime formation by diatoms and exorbitant cost [131]. With nanotechnology, it is possible to combine the knowledge of QQ compounds and material science to create a more economic and durable anti-biofouling approach.

The colonization of the surface by bacteria initiates the subsequent successional colonization process, in which the colonization by microscopic eukaryotes promotes the settlement of invertebrates, evolving from microfouling to soft macrofouling and lastly macrofouling [134]. Thus, inhibiting the initial stages of colonization, such as bacterial biofilm formation, will prevent macrofouling [131,138]. This colonization model has been challenged. Hence, more research has to be carried out to study the factors that lead to the attachment of microorganisms and other larger organisms.

Besides marine biofouling, membrane biofouling has been one of the major problems in industrial processes such as membrane bioreactor and the reverse osmosis/nanofiltration process. Several approaches, such as ozonation [139], nitric oxide [140], enzymatic disruption of extracellular polysaccharide matrix [141] and the modification of the membrane surface [142], have been tried to mitigate biofouling. The use of QQ compounds as a biofouling-control agent has been reported by Yeon *et al.* [91], who later successfully used “acylase I-immobilized nanofiltration membrane” to mitigate biofouling [92,143].

### 4.3. Quorum Quenching in Aquaculture

The ubiquitous use of antibiotics in the aquaculture industry to eliminate pathogens leads to the prevalence of antibiotic resistant bacteria [144]. QQ compounds can be used to control pathogens. Pathogens can also be controlled by the biocontrol approach, which is planting QQ enzyme-producing or QQ compound-producing organisms, such as algae and sponges, in the hatchery [145]. The review by Natrah *et al.* has documented several types of QQ compounds produced by aquatic organisms and their effects on the QS mechanism [146]. Another approach in controlling aquaculture-related diseases is via the use of probiotic bacteria such as *Bacillus* [147,148]. Hitherto, mounting evidence has indicated the feasibility of applying QQ in aquaculture and the outcomes illustrate the effectiveness of this QQ approach [149–151].

### 4.4. Quorum Quenching in Agriculture

Phytopathogens, such as *Xanthomonas* sp., *P. syringae* and *Pectobacterium atrosepticum* (*Erwinia carotovora* subsp. *atroseptica*), and the failure of current bactericides have caused tremendous losses in crop production. They raise an issue in the protection of crops from microbial diseases: are we losing the battle against phytopathogens? Fighting phytopathogens is still the global challenge since the agricultural revolution. One of the potential solutions to this problem is to use the QQ approach, such as using QQ bacteria as biocontrol agents, QQ compounds which attenuate the QS mechanism, or QQ enzymes which inactivate the signalling molecules of phytopathogens. Transgenic potato and tobacco plants expressing QQ enzymes (e.g., lactonases) are protected against Gram-negative phytopathogens such as *E. carotovora* [30]. This is one of the successful examples in applying the QQ approach to control bacterial infection. The drawback of this approach is the adoption of transgenic technology which is not universally accepted and involves biosafety issues as regulated by the Cartagena Protocol. A possible way of applying the QQ approach in agriculture would be to use QQ bacteria as biocontrol agents. For example, *B. thuringiensis*, which is well known for its efficient QQ activity, can be used to control bacterial infection [115]. *B. thuringiensis* would be an attractive QQ biocontrol agent in modern industrial agriculture where monoculture is practiced.

## 5. Quorum Quenching Resistance: Final Destination?

Since the discovery of penicillin by Alexander Fleming in 1928, more antibiotics have been discovered and developed, thereby revolutionizing medicine by increasing the average life expectancy of humankind. In 1940s, the first report of penicillin resistant *Staphylococcus aureus* marked the beginning of the battle between mankind and bacteria [152]. In 2004, more than 70% of pathogenic bacteria have been predicted to be resistant to at least one of the currently available antibiotics [153]. The rapid emergence of multidrug resistant bacterial pathogens, such as methicillin-resistant *S. aureus* (MRSA), *Escherichia coli* strain O104 and *K. pneumonia* harboring NDM-1 (New Delhi metallo-β-lactamase), illustrates the limitation of antibiotics.

QQ may pose lesser or no evolutionary pressure on pathogenic bacteria, thereby reducing the chances of them emerging as multidrug resistant strains [154]. More translational medicinal research should be carried out to investigate the possibility of putting QQ compounds and enzymes into applications. Several reports on the application of this approach in anti-virulence are very convincing [155,156]. It is encouraging to notice numerous QQ compounds have been discovered and studied, and some even used in clinical trial [157,158]. This may make QQ an effective approach to combat bacterial diseases.

However, this idea has been challenged by Defoirdt *et al.* who suggested that variations in the expression of QS core genes (*i.e.*, genes involved in the signal synthesis, detection and transduction) might lead to differences in fitness and natural selection might favour mutants resistant to QS disruption [159]. In 2010, Köhler *et al.* reported that treatment with a QQ compound (azithromycin) selects for QS-proficient strain and increases the prevalence of virulent *P. aeruginosa* [160]. They cautioned the use of QQ compounds as anti-virulence drugs and highlighted the need to assess the impact of intervention on the evolution of virulence of pathogenic bacteria [160].

Recently, Maeda *et al.* provided evidence of bacterial resistance to anti-virulence compounds [161]. They used a random transposon mutagenesis approach to screen for mutants capable of growing on minimal medium containing adenosine as the sole carbon source, in the presence of brominated furanone C-30, a QQ compound. Mutations were found in *mexR* and *nalC*. The *mexR* gene, responsible for the down regulation of the *mexAB-oprM* operon, encodes a multidrug efflux system that contributes to intrinsic and acquired multidrug resistance [162]. *nalC* (also known as *PA3721*) encodes a repressor for TetR/AcrR. Mutation in *nalC* leads to the expression of a two-gene operon namely *PA3720-PA3719*, and PA3719 (also known as ArmR) will form a complex with MexR, reducing the repressor activity of MexR [163,164]. Maeda *et al.* also isolated *P. aeruginosa* with mutations in *mexR* and *nalC* from cystic fibrosis patients who have undergone antibiotic therapy. They postulated that inactivation of *mexR* leads to the expression of *mexAB-oprM* operon, thereby enhancing the efflux of C-30 by the *P. aeruginosa* cells. This serves as the defense mechanism of bacteria against antibiotics and QQ compounds, which exert a selective pressure on bacteria. There is a possibility that antibiotic treatments induce resistance to anti-virulence QQ compounds, or vice versa, as mutations that occur are targeted at the *mexAB-oprM* operon which plays an important role in the efflux of antibiotics and probably C-30, in order to regain the selective advantage [161].

Several reports have shown that it is possible for bacteria to escape from the QQ effect by regulating or altering their genetic circuits. For example, point mutations at the LuxR homolog signal binding site may cause the receptor to become insensitive to its antagonist, or to turn antagonist into agonist, activating the QS pathway [23]. This suggests that variation in the structure or sequence of the LuxR homolog signal binding site may lead to inhibitor resistance.

While various bacterial strains produce different signalling molecules, some bacteria never produce signalling molecules [165]. Furthermore, variation in the concentration of signal receptors may affect the effectiveness in the inhibitory effect [159]. For example, overexpression of LuxR homolog in *A. tumefaciens*, *i.e.*, TraR, results in the failure of analogs to inhibit the QS mechanism [166]. The variation in the concentration of signal receptor might be crucial considering the competition between QS antagonist and the signalling molecules for the binding of receptor. Also, different types of signalling molecules produced have different affinities towards the cognate LuxR receptor [167]. This has been a challenge in developing approaches that target a broad range of signalling molecules.

Variation in the number of LuxI and LuxR homologs between different strains of the same species has been reported for several bacteria, such as *Burkholderia mallei, Burkholderia pseudomallei* and *Rhizobium etli*, in which the number of LuxR homolog ranges from two to nine [168]. This condition poses a challenge for the design of receptor-binding antagonists. Furthermore, some of the LuxR homologs are orphans, *i.e.*, *luxR* homologs that are not linked to or associated with an AHL-synthase-encoding *luxI* in the bacterial genomes. This raises questions regarding the possible roles of these orphan LuxR homologs and the possibility of these orphan LuxR homologs modulating genes that are regulated by the cognate *luxR/I* pair [169]. Furthermore, compensatory mutation, which leads to the restoration of social independence by playing a role as a “cooperator” (QS-proficient) instead of “cheater” (QS-deficient), has been proposed as one of the possible mechanisms of bacteria to overcome the interference of QS and this might lead to the development of resistance against interference of QS [170–172].]. This condition has been observed in *P. aeruginosa* by Dekimpe and Déziel in which RhlR can overcome the absence of *las* system by regulating LasR-specific factors [173]. One of the explanations given is the binding of signalling molecule (3-oxo-C12-HSL) to another QS regulator, *i.e.*, QscR. This binding phase-differentially activates and represses *lasR* and *rhlR*, thereby affecting the expression of downstream gene loci [174]. A recent finding shows that mutation in Qrr sRNA in *Vibrio cholerae* leads to the failure of Qrr/*hapR* binding (*hapR* is the gene that encodes LuxR homolog in *V. cholera*), thereby causing the restoration of HapR function which activates and represses multiple genes activities [175]. But a mutant possessing only one of the four Qrr sRNAs is able to engage QS-related activities [175], making this challenging for receptor-binding antagonists to act against *V. cholera* QS system.

Variation in the spatial arrangement of *luxI* and *luxR* homologs might alter their expression and cause certain QS-regulated traits to fail to be controlled by the QS mechanism [159]. The locations of the *traIR* and *smaIR* operons on the Ti plasmid of *A. tumefaciens* and a transposon in *Serratia marcescens*, respectively, may enable them to shun from the disruption of their innate QS mechanism owing to horizontal gene transfer and transposition [176,177].

QQ compounds have been shown to increase clearance of bacteria by the host's immune system and decrease colonization [178,179]. Hence, QQ compounds lead to a decrease in fitness and mutants that are insensitive to the QQ compounds gain a selective advantage over the QQ compound-sensitive wild type [159]. Treating a population of QS-proficient bacteria with QQ compounds might yield two possibilities. First, it might shut down QS pathways, causing pathogens to fail to produce QS-regulated extracellular virulence factors. In this scenario, the bacteria behave like the “cheater” as described elsewhere [180,181]. Second, evolution might occur in a small proportion of the population and QQ compound-insensitive mutants might continue to constitutively express QS-regulated genes. In a mixture containing the wild type and mutant bacteria exposed to a QQ compound, the latter will produce QS-regulated enzymes to breakdown environmental macromolecules into simpler form (public good). The former will be exploiting the public good. In this scenario the fitness advantage of the QQ compound-resistant mutant decreases as compared with the QQ compound-sensitive wild type owing to competition for nutrients. Hence, it has been suggested that social cheating would play an important role in slowing down the development of resistance to QQ compounds [182]. When the QQ compound is removed, the wild type will regain its ability to produce signalling molecule(s) together with the QQ compound-resistant-mutant. The bacterial population will reach the threshold value of signalling molecules rapidly. Hence, it appears that a single QQ compound is not suitable to be used therapeutic purpose as its discontinuity in patients may cause to them to be infected by highly virulent bacteria.

In most experiments, bacteria are cultured on nutrient-rich growth media. In these conditions no selective pressure is exerted and QS-regulated traits are not essential for survival. Hence, testing the QQ compounds *in vivo*, especially during infection, to investigate the association between the targeted QQ compounds and the fitness of bacteria will allow us to anticipate the possibility of QQ resistance development. Besides, different nutrients will be available for *in vivo* testing and the metabolism of some of them might not be regulated by QS pathways [159]. Thus, *in vivo* testing of QQ compounds will allow us to examine traits that are not regulated by QS and the possibility of development of QQ resistance.

Several strategies to reduce the risk of QQ resistance development have been recommended [159]. The first approach is the use of QQ enzymes that target a broad range of AHLs. Several QQ enzymes, such as lactonase and acylase are able to inactivate a wide range of AHLs. However, this approach of targeting various AHLs raises concern over the consequences of inactivating beneficial microbial QS pathways. The interference of host microflora and its implications should be considered. Another challenge would be the stability of QQ enzymes in various physical and chemical conditions and in the host. The advantage of this approach is that it is not targeting the LuxR homolog or any receptor but the signalling molecules. Thus, this approach is still feasible even if there are mutations at the AHL binding site of the LuxR homologs. This approach also prevents the binding of AHLs by inactivating them, unless they have a structure that is not recognized by the QQ enzymes. The second approach involves the use of different QQ compounds against bacteria. In view of the existence of some bacteria resistant to existing QQ compounds, much like the fate of antibiotics, new QQ compounds are needed to suppress the virulence of bacteria. The third approach combines the QQ approach with other treatments, such as antibiotics, to obtain a synergistic effect [130], in which the QQ compound disarms and increases the susceptibility of bacteria for antibiotic treatment. It remains to be seen whether this “cocktail” therapy of antibiotic and QQ compounds will lead to resistance to both chemicals. The fourth and last approach targets the virulence factors. This approach expands the repertoire of bacterial targets, aims to preserve the host endogenous microflora and imposes a relatively mild selective pressure which may slow down or circumvent the development of resistance [183].

## 6. Conclusions/Outlook

As antibiotic resistant bacteria become a global threat to public health, novel therapeutics represent an important area of current scientific research. Attenuating virulence without causing selection pressure is an attractive approach. QS is a key regulatory system that controls the expression of virulence determinants, thus making QS an effective target for novel drug design as well as agricultural and industrial applications. QQ provides a strategy to disrupt QS, and in turn attenuates virulence determinants. The future of QS research lies in the discovery of additional communication signals and their *modus operandi*. Consequently, QQ as a promising anti-infective strategy can be developed based on information obtained from QS studies. This suggests that QS and QQ will continue to assert impact on microbial communication research.

As a concluding remark, while research on bacterial QS has led to discovery of promising targets for novel anti-infective therapy, and eukaryotes including plants have emerged as new sources of QQ compounds that may be explored for biotechnological and pharmaceutical applications, we must be cautious messing with bacterial QS system so as not to open another Pandora's box.

## Figures and Tables

**Table 1.** Characterization of various microbial AHL-degrading enzymes.

**Lactonase Activity**									
**Strain or Source**	**Taxonomy (Class)**	**Name of QQ Enzyme**	**Protein Family**	**Zinc Binding Motif**	**AHL Degradation**	**Number of Amino Acids**	**Protein Size (kDa)**	**Metal Ion Required**	**Reference**
*Agrobacterium tumefaciens* c58	Proteobacteria	AttM	Metallo-β-lactamase superfamily	HXHXDH	3-oxo-C8-HSL, C6-HSL	263	29	Zn^2+^	[48]
*Arthrobacter* sp. IBN110	Actinobacteria	AhlD	Metallo-β-lactamase superfamily	HXHXDH	3-oxo-C6-HSL, C4-HSL, C6-HSL, C8-HSL, C10-HSL	273	31	Zn^2+^	[50]
*Bacillus* sp. 240B1	Firmicutes	AiiA	Metallo-β-lactamase superfamily	HXHXDH	3-oxo-C6-HSL, 3-oxo-C8-HSL, 3-oxo-C10-HSL	250	28	Zn^2+^	[31]
*Geobacillus kaustophilus* strain HTA426	Firmicutes	GKL	Amidohydrolase superfamily	nd.	C6-HSL, C8-HSL, C10-HSL, 3-oxo-C8-HSL and 3-oxo-C12-HSL	330	37	Zn^2+^	[43]
*Microbacterium testaceum* StLB037	Actinobacteria	AiiM	α/β hydrolase fold family	nd.	3-oxo-C6-HSL, C6-HSL, 3-oxo-C8-HSL, C8-HSL, 3-oxo-C10-HSL, C10-HSL	251	27	None	[52]
*Mycobacterium avium* subsp. *paratuberculosis* K-10	Actinobacteria	MCP	Amidohydrolase superfamily	nd.	C7-HSL, C8-HSL, 3-oxo-C8-HSL, C10-HSL, C12-HSL	326	nd.	Mn^2+^	[47]
*Mycobacterium tuberculosis*	Actinobacteria	PPH	Amidohydrolase superfamily	nd.	C4-HSL, C10-HSL, 3-oxo-C8-HSL	326	nd.	Mn^2+^	[45]
*Ochrobactrum* sp. T63	Proteobacteria	AidH	α/β hydrolase fold family	nd.	C4-HSL, C6-HSL, 3-oxo-C6-HSL, 3-oxo-C8-HSL, C10-HSL	271	29.5	Mn^2+^	[51]
*Rhodococcus erythropolis* W2	Actinobacteria	QsdA (also known as AhlA)	PTE superfamily	PTE domain	AHLs with or without substitution on carbon 3 and with an acyl chain ranging from 6 to 14 carbons	323	36	Zn^2+^	[45,55]
*Solibacillus silvestris* StLB046	Firmicutes	AhlS	Metallo-β-lactamase superfamily	HXHXDH	C6-HSL, 3-oxo-C6-HSL, C10-HSL, 3-oxo-C10-HSL	277	31	Zn^2+^	[53]
*Sulfolobus solfataricus* strain P2	Thermoprotei (Superkingdom: Archaea)	SsoPox	Amidohydrolase superfamily	PTE domain	3-oxo-C8-HSL, C8-HSL, 3-oxo-C10-HSL, 3-oxo-C12-HSL	314	48	Co^2+^ & Fe^3+^	[61,62]
Soil metagenomic clone	nd.	QlcA	Metallo-β-lactamase superfamily	HXHXDH	C6-HSL, C7-HSL, C8-HSL, C10-HSL, 3-hydroxy-C6-HSL, 3-oxo-C8-HSL, 3-hydroxy-C8-HSL	221	24	Zn^2+^	[63]
Soil metagenomic clone	nd.	BpiB01	Hypothetical protein family	None	3-oxo-C8-HSL	400	45	Zn^2+^	[64]
Soil metagenomic clone	nd.	BpiB04	Glycosyl hydrolase family	None	3-oxo-C8-HSL	135	18	Zn^2+^	[64]
Soil metagenomic clone	nd.	BpiB05	Dienelactone hydrolase family	None	3-oxo-C8-HSL	587	70	Zn^2+^	[65]
Soil metagenomic clone	nd.	BpiB07	Hypothetical protein family	None	3-oxo-C8-HSL	265	29	Ca^2+^	[64]
**Acylase Activity**									
**Strain or Source**	**Taxonomy (Class)**	**Name of QQ Enzyme**	**Protein Family**	**Zinc Binding Motif**	**AHL Degradation**	**Number of Amino Acids**	**Protein Size (kDa)**	**Metal Ion Required**	**Reference**
*Anabaena* sp. PCC7120	Cyanobacteria	AiiC	nd.	nd.	AHLs with or without substitution on carbon 3 and with an acyl chain ranging from 4 to 14 carbons	847	nd.	nd.	[66]
*Comamonas* sp. strain D1	Proteobacteria	nd.	nd.	nd.	AHLs with or without substitution on carbon 3 and with an acyl chain ranging from 4 to 16 carbons	nd.	nd.	nd.	[67]
*Pseudomonas aeruginosa* PAO1	Proteobacteria	PvdQ	Ntn-hydrolase	nd.	AHLs with or without substitution on carbon 3 and with an acyl chain ranging from 10 to 14 carbons	726	18 kDa (α-subunit); 60 kDa (β-subunit)	nd.	[68,69]
*Pseudomonas aeruginosa* PAO1	Proteobacteria	QuiP	Ntn-hydrolase	nd.	AHLs with or without substitution on carbon 3 and with an acyl chain ranging from 7 to 14 carbons	847	90	nd.	[70]
*Pseudomonas syringae* strain B728a	Proteobacteria	HacA	Ntn-hydrolase	nd.	C8-HSL, C10-HSL and C12-HSL	779	85	nd.	[71]
*Pseudomonas syringae* strain B728a	Proteobacteria	HacB	Ntn-hydrolase	nd.	AHLs with or without substitution on carbon 3 and with an acyl chain ranging from 6 to 12 carbons	795	88	nd.	[71]
*Ralstonia* sp. XJ12B	Proteobacteria	AiiD	Ntn-hydrolase	nd.	3-oxo-C8-HSL, 3-oxo-C10-HSL and 3-oxo-C12-HSL (less activity against 3-oxo-C6-HSL)	794	nd.	nd.	[32]
*Ralstonia solanacearum* GMI1000	Proteobacteria	Aac	nd.	nd.	C7-HSL and C8-HSL, 3-oxo-C8-HSL and C10-HSL	824	88	nd.	[72]
*Rhodococcus erythropolis* W2	Actinobacteria	nd.	nd.	nd.	3-oxo-C10-HSL	nd.	nd.	nd.	[73]
*Shewanella* sp. strain MIB015	Proteobacteria	Aac	nd.	nd.	C8-HSL, C10-HSL and C12-HSL	855	nd.	nd.	[74]
*Streptomyces* sp. strain M664	Actinobacteria	AhlM	Ntn-hydrolase	nd.	C8-HSL, C10-HSL, 3-oxo-C12-HSL	804	23 kDa and 60 kDa (2 subunits)	nd.	[75]
*Tenacibaculum maritimum* strain NCIMB2154(T)	Bacteroidetes	nd.	nd.	nd.	C10-HSL	nd.	nd.	nd.	[76]
*Variovorax paradoxus* VAI-C	Proteobacteria	nd.	nd.	nd.	C4-HSL, C6-HSL, 3-oxo-C6-HSL, C8-HSL, C10-HSL, C12-HSL, C14-HSL	nd.	nd.	nd.	[77]
**Oxidoreductase Activity**									
**Strain or Source**	**Taxonomy (Class)**	**Name of QQ Enzyme**	**Protein Family**	**Zinc Binding Motif**	**AHL Degradation**	**Number of Amino Acids**	**Protein Size (kDa)**	**Metal Ion Required**	**Reference**
*Bacillus megaterium* CYP102A1	Firmicutes	nd.	nd.	nd.	Oxidizes C12-HSL to C20-HSL to corresponding ω-1, ω-2 and/ or ω-3 hydroxylated AHLs.	nd.	nd.	nd.	[78]
*Burkholderia* sp. strain GG4	Proteobacteria	nd.	nd.	nd.	Reduces 3-oxo-AHL to corresponding 3-hydroxy derivatives	nd.	nd.	nd.	[57]
*Rhodococcus erythropolis* W2	Actinobacteria	nd.	nd.	nd.	Converts C8-HSL to C14-HSL to corresponding 3-hydroxy derivatives	nd.	nd.	nd.	[73]
Soil metagenomic clone	nd.	BpiB09	Short-chain reductases	nd.	Reduces 3-oxo-C12-HSL to 3-hydroxy-C12-HSL	nd.	27.4	nd.	[79]
**Activity Not Determined**									
**Strain or Source**	**Taxonomy (Class)**	**Name of QQ Enzyme**	**Protein Family**	**Zinc Binding Motif**	**AHL Degradation**	**Number of Amino Acids**	**Protein Size (kDa)**	**Metal Ion Required**	**Reference**
*Acinetobacter* sp. strain C1010	Proteobacteria	nd.	nd.	nd.	nd.	nd.	nd.	nd.	[56]
*Delftia* sp.	Proteobacteria	nd.	nd.	nd.	nd.	nd.	nd.	nd.	[80]

**Table 2.** The roles of quorum quenching enzymes.

**Organism**	**Role of Quorum Quenching Enzyme**	**Reference**
*Agrobacterium tumefaciens* c58	AiiB modulates the conjugation frequency of Ti plasmid and the emergence of tumour; AttM enhances the fitness of *A. tumefaciens* in the plant tumour	[48,49,93]
	BlcC (or AttM) metabolizes GBL, yielding succinic acid for Krebs cycle	[94]
*Anabaena* (Nostoc) sp. PCC 7120	Interference with the communication system within the complex microbial communities	[66]
	Control of the cytotoxicity effect of AHLs	[95]
*Arthrobacter* sp. IBN 110	Metabolism of AHLs as carbon and nitrogen sources	[50]
*Bacillus* sp.	Microbial competition	[96]
	Control of the toxicity effects of AHLs and tetramic acid derivatives; Competition for iron from the environment	[97]
*Bacillus megaterium* CYP102A1	Interference with QS pathways; Prevention of the accumulation of degraded AHL products; Enhancement of diffusion away from the cell	[78]
*Burkholderia* sp. strain GG4	Unknown	[57]
*Chryseobacterium* sp.	Utilization of AHL degradation products as carbon and nitrogen sources; Providing protection to the plant from pathogens for the purpose of symbiotic interaction with the host	[59]
*Geobacillus caldoxylosilyticus* YS-8	Unknown	[44]
*Geobacillus kaustophilus* HTA426	Unknown	[43]
*Microbacterium testaceum* StLB037	Providing protection to the plant from pathogens for the purpose of symbiotic interaction with the host	[52]
*Mycobacterium avium* subsp. *paratuberculosis* K-10	Unknown	[47]
*Nocardioides kongjuensis* strain A2-4^T^	Metabolism of AHLs as carbon and nitrogen sources	[58]
*Ochrobactrum* sp. strain T63	Unknown	[51]
*Pseudomonas aeruginosa* PAO1	Regulation of pyoverdine biosynthesis	[98]
	Utilization of AHL; involvement in the maturation of pyoverdine siderophore; regulation of 3-oxo-C12-HSL	[68]
	Regulation of virulence phenotype	[69]
	Playing a role in iron sequestration	[99]
	Regulation of flagellum-dependent motions; Development of antibiotic resistance	[100]
	Development of pyoverdine I	[101]
*Ralstonia solanacearum* GMI1000	Metabolism of AHL as source of carbon and nitrogen	[72]
	Modulation of the QS pathways or as a signal turnover mechanism	[32]
*Ralstonia* strain JX12B	Oligotrophic nutrient scavenging from the natural environment	[32]
*Rhodococcus erythropolis* W2	Probable involvement in fatty acid metabolism	[55]
*Shewanella* sp strain MIB015	Unknown	[74]
*Streptomyces* sp. strain M664	Unknown	[75]
*Variovorax paradoxus* VAI-C	Metabolism of AHLs as carbon and nitrogen sources	[77]

## References

[1] Fuqua W.C., Winans S.C., Greenberg E.P. (1994). Quorum sensing in bacteria: The LuxR-LuxI family of cell density-responsive transcriptional regulators. J. Bacteriol..

[2] Fuqua C., Parsek M.R., Greenberg E.P. (2001). Regulation of gene expression by cell-to-cell communication: Acyl-homoserine lactone quorum sensing. Annu. Rev. Genet..

[3] Williams P., Winzer K., Chan W.C., Cámara M. (2007). Look who's talking: Communication and quorum sensing in the bacterial world. Phil. Trans. R. Soc. B..

[4] Eberhard A., Burlingame A.L., Eberhard C., Kenyon G.L., Nealson K.H., Oppenheimer N.J. (1981). Structural identification of autoinducer of *Photobacterium fischeri* luciferase. Biochemistry.

[5] Chen X., Schauder S., Potier N., Van Dorsselaer A., Pelczer I., Bassler B.L., Hughson F.M. (2002). Structural identification of a bacterial quorum-sensing signal containing boron. Nature.

[6] Zhu J., Dizin E., Hu X., Wavreille A.S., Park J., Pei D. (2003). *S*-Ribosylhomocysteinase (LuxS) is a mononuclear iron protein. Biochemistry.

[7] Flavier A.B., Ganova-Raeva L.M., Schell M.A., Denny T.P. (1997). Hierarchical autoinduction in *Ralstonia solanacearum*: Control of acyl-homoserine lactone production by a novel autoregulatory system responsive to 3-hydroxypalmitic acid methyl ester. J. Bacteriol..

[8] Wang L.H., He Y., Gao Y., Wu J.E., Dong Y.H., He C., Wang S.X., Weng L.X., Xu J.L., Tay L. (2004). A bacterial cell-cell communication signal with cross-kingdom structural analogues. Mol. Microbiol..

[9] Ohnishi Y., Kameyama S., Onaka H., Horinouchi S. (1999). The A-factor regulatory cascade leading to streptomycin biosynthesis in *Streptomyces griseus*: Identification of a target gene of the A-factor receptor. Mol. Microbiol..

[10] Holden M.T., Ram Chhabra S., de Nys R., Stead P., Bainton N.J., Hill P.J., Manefield M., Kumar N., Labatte M., England D. (1999). Quorum-sensing cross talk: Isolation and chemical characterization of cyclic dipeptides from *Pseudomonas aeruginosa* and other Gram-negative bacteria. Mol. Microbiol..

[11] Diggle S.P., Winzer K., Chhabra S.R., Worrall K.E., Cámara M., Williams P. (2003). The *Pseudomonas aeruginosa* quinolone signal molecule overcomes the cell density-dependency of the quorum sensing hierarchy, regulates *rhl*-dependent genes at the onset of stationary phase and can be produced in the absence of LasR. Mol. Microbiol..

[12] Déziel E., Lépine F., Milot S., He J., Mindrinos M.N., Tompkins R.G., Rahme L.G. (2004). Analysis of *Pseudomonas aeruginosa* 4-hydroxy-2-alkylquinolines (HAQs) reveals a role for 4-hydroxy-2-heptylquinoline in cell-to-cell communication. Proc. Natl. Acad. Sci. USA.

[13] Ji G., Beavis R.C., Novick R.P. (1995). Cell density control of staphylococcal virulence mediated by an octapeptide pheromone. Proc. Natl. Acad. Sci. USA.

[14] Dong Y.H., Wang L.H., Zhang L.H. (2007). Quorum sensing microbial infections: Mechanisms and implications. Phil. Trans. R. Soc. B.

[15] Miller M.B., Bassler B.L. (2001). Quorum sensing in bacteria. Annu. Rev. Microbiol..

[16] Decho A.W., Frey R.L., Ferry J.L. (2011). Chemical challenges to bacterial AHL signalling in the environment. Chem. Rev..

[17] Paggi R.A., Martone C.B., Fuqua C., De Castro R.E. (2003). Detection of quorum sensing signals in the haloalkaliphilic archaeon *Natronococcus occultus*. FEMS Microbiol. Lett..

[18] Farah C., Vera M., Morin D., Haras D., Jerez C.A., Guiliani N. (2005). Evidence for a functional quorum-sensing type AI-1 system in the extremophilic bacterium *Acidithiobacillus ferrooxidans*. Appl. Environ. Microbiol..

[19] Rivas M., Seeger M., Jedlicki E., Holmes D.S. (2007). Second acyl homoserine lactone production system in the extreme acidophile *Acidithiobacillus ferrooxidans*. Appl. Environ. Microbiol..

[20] Soulère L., Guiliani N., Queneau Y., Jerez C.A., Doutheau A. (2008). Molecular insights into quorum sensing in *Acidithiobacillus ferrooxidans* bacteria via molecular modelling of the transcriptional regulator AfeR and of the binding mode of long-chain acyl homoserine lactones. J. Mol. Model..

[21] Sharif D.I., Gallon J., Smith C.J., Dudley E. (2008). Quorum sensing in cyanobacteria: *N*-octanoyl-homoserine lactone release and response, by the epilithic colonial cyanobacterium *Gloeothece* PCC6909. ISME J..

[22] Parveen N., Cornell K.A. (2011). Methylthioadenosine/*S*-adenosylhomocysteine nucleosidase, a critical enzyme for bacterial metabolism. Mol. Microbiol..

[23] Koch B., Liljefors T., Persson T., Nielsen J., Kjelleberg S., Givskov M. (2005). The LuxR receptor: The sites of interaction with quorum-sensing signals and inhibitors. Microbiology.

[24] Chen G., Swern L.R., Swern D.L., Stauff D.L., O'Loughlin C.T., Jeffrey P.D., Bassler B.L., Hughson F.M. (2011). A strategy for antagonizing quorum sensing. Mol. Cell..

[25] Manefield M., de Nys R., Kumar N., Read R., Givskov M., Steinberg P., Kjelleberg S. (1999). Evidence that halogenated furanones from *Delisea pulchra* inhibit acylated homoserine lactone (AHL)-mediated gene expression by displacing the AHL signal from its receptor protein. Microbiology.

[26] Geske G.D., O'Neill J.C., Blackwell H.E. (2008). Expanding dialogues: From natural autoinducers to non-natural analogues that modulate quorum sensing in Gram-negative bacteria. Chem. Soc. Rev..

[27] Galloway W.R., Hodgkinson J.T., Bowden S.D., Welch M., Spring D.R. (2011). Quorum sensing in Gram-negative bacteria: Small-molecule modulation of AHL and AI-2 quorum sensing pathways. Chem. Rev..

[28] Stevens A.M., Queneau Y., Soulère L., von Bodman S., Doutheau A. (2011). Mechanisms and synthetic modulators of AHL-dependent gene regulation. Chem. Rev..

[29] Dong Y.H., Zhang L.H. (2005). Quorum sensing and quorum-quenching enzymes. J. Microbiol..

[30] Dong Y.H., Wang L.H., Xu J.L., Zhang H.B., Zhang X.F., Zhang L.H. (2001). Quenching quorum-sensing-dependent bacterial infection by an *N*-acyl homoserine lactonase. Nature.

[31] Dong Y.H., Xu J.L., Li X.Z., Zhang L.H. (2000). AiiA, an enzyme that inactivates the acylhomoserine lactone quorum-sensing signal and attenuates the virulence of *Erwinia carotovora*. Proc. Natl. Acad. Sci. USA.

[32] Lin Y.H., Xu J.L., Hu J., Wang L.H., Ong S.L., Leadbetter J.R., Zhang L.H. (2003). Acyl-homoserine lactone acylase from *Ralstonia* strain XJ12B represents a novel and potent class of quorum-quenching enzymes. Mol. Microbiol..

[33] Daiyasu H., Osaka K., Ishino Y., Toh H. (2001). Expansion of the zinc metallo-hydrolase family of the beta-lactamase fold. FEBS Lett..

[34] Kim M.H., Choi W.C., Kang H.O., Lee J.S., Kang B.S., Kim K.J., Derewenda Z.S., Oh T.K., Lee C.H., Lee J.K. (2005). The molecular structure and catalytic mechanism of a quorum-quenching *N*-acyl-L-homoserine lactone hydrolase. Proc. Natl. Acad. Sci. USA.

[35] Wang L.H., Weng L.X., Dong Y.H., Zhang L.H. (2004). Specificity and enzyme kinetics of the quorum-quenching *N*-acyl homoserine lactone lactonases (AHL-lactonase). J. Biol. Chem..

[36] Liu D., Lepore B.W., Petsko G.A., Thomas P.W., Stone E.M., Fast W., Ringe D. (2005). Three-dimensional structure of the quorum-quenching *N*-acyl homoserine lactone hydrolase from *Bacillus thuringiensis*. Proc. Natl. Acad. Sci. USA.

[37] Thomas P.W., Stone E.M., Costello A.L., Tierney D.L., Fast W. (2005). The quorum-quenching lactonase from *Bacillus thuringiensis* is a metalloprotein. Biochemistry.

[38] Momb J., Wang C., Liu D., Thomas P.W., Petsko G.A., Guo H., Ringe D., Fast W. (2008). Mechanism of the quorum-quenching lactonase (AiiA) from *Bacillus thuringiensis*. 2. Substrate modeling and active site mutations. Biochemistry.

[39] Lu X., Yuan Y., Xue X.L., Zhang G.P., Zhou S.N. (2006). Identification of the critical role of Tyr-194 in the catalytic activity of a novel *N*-acyl-homoserine lactonase from marine *Bacillus cereus* strain Y2. Curr. Microbiol..

[40] Huma N., Shankar P., Kushwah J., Bhushan A., Joshi J., Mukherjee T., Raju S., Purohit H., Kalia V. (2011). Diversity and polymorphism in AHL-lactonase gene (*aiiA*) of *Bacillus*. J. Microbiol. Biotechnol..

[41] Liao R.Z., Yu J.G., Himo F. (2009). Reaction mechanism of the dinuclear zinc enzyme *N*-acyl-l-homoserine lactone hydrolase: A quantum chemical study. Inorg. Chem..

[42] Dong Y.H., Gusti A.R., Zhang Q., Xu J.L., Zhang L.H. (2002). Identification of quorum-quenching *N*-acyl homoserine lactonases from *Bacillus* species. Appl. Environ. Microbiol..

[43] Chow J.Y., Xue B., Lee K.H., Tung A., Wu L., Robinson R.C., Yew W.S. (2010). Directed evolution of a thermostable quorum-quenching lactonase from the amidohydrolase superfamily. J. Biol. Chem..

[44] Seo M.J., Lee B.S., Pyun Y.R., Park H. (2011). Isolation and characterization of *N*-acylhomoserine lactonase from the thermophilic bacterium, *Geobacillus caldoxylosilyticus* YS-8. Biosci. Biotechnol. Biochem..

[45] Afriat L., Roodveldt C., Manco G., Tawfik D.S. (2006). The latent promiscuity of newly identified microbial lactonases is linked to a recently diverged phosphotriesterase. Biochemistry.

[46] Hawwa R., Aikens J., Turner R.J., Santarsiero B.D., Mesecar A.D. (2009). Structural basis for thermostability revealed through the identification and characterization of a highly thermostable phosphotriesterase-like lactonase from *Geobacillus stearothermophilus*. Arch. Biochem. Biophys..

[47] Chow J.Y., Wu L., Yew W.S. (2009). Directed evolution of a quorum-quenching lactonase from *Mycobacterium avium* subsp. *paratuberculosis* K-10 in the amidohydrolase superfamily. Biochemistry.

[48] Zhang H.B., Wang L.H., Zhang L.H. (2002). Genetic control of quorum-sensing signal turnover in *Agrobacterium tumefaciens*. Proc. Natl. Acad. Sci. USA.

[49] Haudecoeur E., Tannières M., Cirou A., Raffoux A., Dessaux Y., Faure D. (2009). Different regulation and roles of lactonases AiiB and AttM in *Agrobacterium tumefaciens* C58. Mol. Plant Microbe Interact..

[50] Park S.Y., Lee S.J., Oh T.K., Oh J.W., Koo B.T., Yum D.Y., Lee J.K. (2003). AhlD, an *N*-acylhomoserine lactonase in *Arthrobacter* sp., and predicted homologues in other bacteria. Microbiology.

[51] Mei G.Y., Yan X.X., Turak A., Luo Z.Q., Zhang L.Q. (2010). AidH, an alpha/beta-hydrolase fold family member from an *Ochrobactrum* sp. strain, is a novel *N*-acylhomoserine lactonase. Appl. Environ. Microbiol..

[52] Wang W.Z., Morohoshi T., Ikenoya M., Someya N., Ikeda T. (2010). AiiM, a novel class of *N*-acylhomoserine lactonase from the leaf-associated bacterium *Microbacterium testaceum*. Appl. Environ. Microbiol..

[53] Morohoshi T., Tominaga Y., Someya N., Ikeda T. (2012). Complete genome sequence and characterization of the *N*-acylhomoserine lactone-degrading gene of the potato leaf-associated *Solibacillus silvestris*. J. Biosci. Bioeng..

[54] Park S.Y., Hwang B.J., Shin M.H., Kim J.A., Kim H.K., Lee J.K. (2006). *N*-acylhomoserine lactonase producing *Rhodococcus* spp. with different AHL-degrading activities. FEMS Microbiol. Lett..

[55] Uroz S., Oger P.M., Chapelle E., Adeline M.T., Faure D., Dessaux Y. (2008). A *Rhodococcus qsdA*-encoded enzyme defines a novel class of large-spectrum quorum-quenching lactonases. Appl. Environ. Microbiol..

[56] Kang B.R., Lee J.H., Ko S.J., Lee Y.H., Cha J.S., Cho B.H., Kim Y.C. (2004). Degradation of acyl-homoserine lactone molecules by *Acinetobacter* sp. strain C1010. Can. J Microbiol..

[57] Chan K.G., Atkinson S., Mathee K., Sam C.K., Chhabra S.R., Cámara M., Koh C.L., Williams P. (2011). Characterization of *N*-acylhomoserine lactone-degrading bacteria associated with the *Zingiber officinale* (ginger) rhizosphere: Co-existence of quorum quenching and quorum sensing in *Acinetobacter* and *Burkholderia*. BMC Microbiol..

[58] Yoon J.H., Lee J.K., Jung S.Y., Kim J.A., Kim H.K., Oh T.K. (2006). *Nocardioides kongjuensis* sp. nov., an *N*-acylhomoserine lactone-degrading bacterium. Int. J. Syst. Evol. Microbiol..

[59] Rashid R., Morohoshi T., Someya N., Ikeda T. (2011). Degradation of *N*-acylhomoserine lactone quorum sensing signalling molecules by potato root surface-associated *Chryseobacterium* strains. Microbes Environ..

[60] d'Angelo-Picard C., Faure D., Penot I., Dessaux Y. (2005). Diversity of *N*-acylhomoserine lactone-producing and -degrading bacteria in soil and tobacco rhizosphere. Environ. Microbiol..

[61] Merone L., Mandrich L., Rossi M., Manco G. (2005). A thermostable phosphotriesterase from the archaeon *Sulfolobus solfataricus*: Cloning, overexpression and properties. Extremophiles.

[62] Elias M., Dupuy J., Merone L., Mandrich L., Porzio E., Moniot S., Rochu D., Lecomte C., Rossi M., Masson P. (2008). Structural basis for natural lactonase and promiscuous phosphotriesterase activities. J. Mol. Biol..

[63] Riaz K., Elmerich C., Raffoux A., Moreira D., Dessaux Y., Faure D. (2008). Metagenomics revealed a quorum quenching lactonase QlcA from yet unculturable soil bacteria. Commun. Agric. Appl. Biol. Sci..

[64] Schipper C., Hornung C., Bijtenhoorn P., Quitschau M., Grond S., Streit W.R. (2009). Metagenome-derived clones encoding two novel lactonase family proteins involved in biofilm inhibition in *Pseudomonas aeruginosa*. Appl. Environ. Microbiol..

[65] Bijtenhoorn P., Schipper C., Hornung C., Quitschau M., Grond S., Weiland N., Streit W.R. (2011). BpiB05, a novel metagenome-derived hydrolase acting on *N*-acylhomoserine lactones. J. Biotechnol..

[66] Romero M., Diggle S.P., Heeb S., Cámara M., Otero A. (2008). Quorum quenching activity in *Anabaena* sp. PCC 7120: Identification of AiiC, a novel AHL-acylase. FEMS Microbiol. Lett..

[67] Uroz S., Oger P., Chhabra S.R., Cámara M., Williams P., Dessaux Y. (2007). *N*-acylhomoserine lactones are degraded via an amidolytic activity in *Comamonas* sp. strain D1. Arch. Microbiol..

[68] Huang J.J., Han J.I., Zhang L.H., Leadbetter J.R. (2003). Utilization of acyl-homoserine lactone quorum signals for growth by a soil pseudomonad and *Pseudomonas aeruginosa* PAO1. Appl. Environ. Microbiol..

[69] Sio C.F., Otten L.G., Cool R.H., Diggle S.P., Braun P.G., Bos R., Daykin M., Cámara M., Williams P., Quax W.J. (2006). Quorum quenching by an *N*-acyl-homoserine lactone acylase from *Pseudomonas aeruginosa* PAO1. Infect. Immun..

[70] Huang J.J., Petersen A., Whiteley M., Leadbetter J.R. (2006). Identification of QuiP, the product of gene PA1032, as the second acyl-homoserine lactone acylase of *Pseudomonas aeruginosa* PAO1. Appl. Environ. Microbiol..

[71] Shepherd R.W., Lindow S.E. (2009). Two dissimilar *N*-acyl-homoserine lactone acylases of *Pseudomonas syringae* influence colony and biofilm morphology. Appl. Environ. Microbiol..

[72] Chen C.N., Chen C.J., Liao C.T., Lee C.Y. (2009). A probable aculeacin A acylase from the *Ralstonia solanacearum* GMI1000 is *N*-acyl-homoserine lactone acylase with quorum-quenching activity. BMC Microbiol..

[73] Uroz S., Chhabra S.R., Cámara M., Williams P., Oger P., Dessaux Y. (2005). *N*-acylhomoserine lactone quorum-sensing molecules are modified and degraded by *Rhodococcus erythropolis* W2 by both amidolytic and novel oxidoreductase activities. Microbiology.

[74] Morohoshi T., Nakazawa S., Ebata A., Kato N., Ikeda T. (2008). Identification and characterization of *N*-acylhomoserine lactone-acylase from the fish intestinal *Shewanella* sp. strain MIB015. Biosci. Biotechnol. Biochem..

[75] Park S.Y., Kang H.O., Jang H.S., Lee J.K., Koo B.T., Yum D.Y. (2005). Identification of extracellular *N*-acylhomoserine lactone acylase from a *Streptomyces* sp. and its application to quorum quenching. Appl. Environ. Microbiol..

[76] Romero M., Avendaño-Herrera R., Magariños B., Cámara M., Otero A. (2010). Acylhomoserine lactone production and degradation by the fish pathogen *Tenacibaculum maritimum*, a member of the Cytophaga-Flavobacterium-Bacteroides (CFB) group. FEMS Microbiol. Lett..

[77] Leadbetter J.R., Greenberg E.P. (2000). Metabolism of acyl-homoserine lactone quorum-sensing signals by *Variovorax paradoxus*. J. Bacteriol..

[78] Chowdhary P.K., Keshavan N., Nguyen H.Q., Peterson J.A., González J.E., Haines D.C. (2007). *Bacillus megaterium* CYP102A1 oxidation of acyl homoserine lactones and acyl homoserines. Biochemistry.

[79] Bijtenhoorn P., Mayerhofer H., Müller-Dieckmann J., Utpatel C., Schipper C., Hornung C., Szesny M., Grond S., Thürmer A., Brzuszkiewicz E. (2011). A novel metagenomic short-chain dehydrogenase/reductase attenuates *Pseudomonas aeruginosa* biofilm formation and virulence on *Caenorhabditis elegans*. PLoS One.

[80] Jafra S., Przysowa J., Czajkowski R., Michta A., Garbeva P., van der Wolf J.M. (2006). Detection and characterization of bacteria from the potato rhizosphere degrading *N*-acyl-homoserine lactone. Can. J. Microbiol..

[81] Ng F.S., Wright D.M., Seah S.Y. (2011). Characterization of a phosphotriesterase-like lactonase from *Sulfolobus solfataricus* and its immobilization for disruption of quorum sensing. Appl. Environ. Microbiol..

[82] Chan K.G., Yin W.F., Sam C.K., Koh C.L. (2009). A novel medium for the isolation of *N*-acylhomoserine lactone-degrading bacteria. J. Ind. Microbiol. Biotechnol..

[83] Nishino S.F., Spain J.C. (2006). Biodegradation of 3-nitrotyrosine by *Burkholderia* sp. strain JS165 and *Variovorax paradoxus* JS171. Appl. Environ. Microbiol..

[84] Wang Y.P., Gu J.D. (2006). Degradability of dimethyl terephthalate by *Variovorax paradoxus* T4 and *Sphingomonas yanoikuyae* DOS01 isolated from deep-ocean sediments. Ecotoxicology.

[85] Snellinx Z., Taghavi S., Vangronsveld J., van der Lelie D. (2003). Microbial consortia that degrade 2,4-DNT by interspecies metabolism: Isolation and characterisation. Biodegradation.

[86] Maskow T., Babel W. (2000). Calorimetrically recognized maximum yield of poly-3-hydroxybutyrate (PHB) continuously synthesized from toxic substrates. J. Biotechnol..

[87] Smith D., Alvey S., Crowley D.E. (2005). Cooperative catabolic pathways within an atrazine-degrading enrichment culture isolated from soil. FEMS Microbiol. Ecol..

[88] Bokhove M., Nadal Jimenez P., Quax W.J., Dijkstra B.W. (2010). The quorum-quenching *N*-acyl homoserine lactone acylase PvdQ is an Ntn-hydrolase with an unusual substrate-binding pocket. Proc. Natl. Acad. Sci. USA.

[89] Uroz S., D'Angelo-Picard C., Carlier A., Elasri M., Sicot C., Petit A., Oger P., Faure D., Dessaux Y. (2003). Novel bacteria degrading *N*-acylhomoserine lactones and their use as quenchers of quorum-sensing-regulated functions of plant-pathogenic bacteria. Microbiology.

[90] Xu F., Byun T., Deussen H.J., Duke K.R. (2003). Degradation of *N*-acylhomoserine lactones, the bacterial quorum-sensing molecules, by acylase. J. Biotechnol..

[91] Yeon K.M., Cheong W.S., Oh H.S., Lee W.N., Hwang B.K., Lee C.H., Beyenal H., Lewandowski Z. (2009). Quorum sensing: A new biofouling control paradigm in a membrane bioreactor for advanced wastewater treatment. Environ. Sci. Technol..

[92] Kim J.H., Choi D.C., Yeon K.M., Kim S.R., Lee C.H. (2011). Enzyme-immobilized nanofiltration membrane to mitigate biofouling based on quorum quenching. Environ. Sci. Technol..

[93] Haudecoeur E., Faure D. (2010). A fine control of quorum-sensing communication in *Agrobacterium tumefaciens*. Commun. Integr. Biol..

[94] Khan S.R., Farrand S.K. (2009). The BlcC (AttM) lactonase of *Agrobacterium tumefaciens* does not quench the quorum-sensing system that regulates Ti plasmid conjugative transfer. J. Bacteriol..

[95] Romero M., Muro-Pastor A.M., Otero A. (2011). Quorum sensing *N*-acylhomoserine lactone signals affect nitrogen fixation in the cyanobacterium *Anabaena* sp. PCC7120. FEMS Microbiol. Lett..

[96] Park S.J., Park S.Y., Ryu C.M., Park S.H., Lee J.K. (2008). The role of AiiA, a quorum-quenching enzyme from *Bacillus thuringiensis*, on the rhizosphere competence. J. Microbiol. Biotechnol..

[97] Kaufmann G.F., Sartorio R., Lee S.H., Rogers C.J., Meijler M.M., Moss J.A., Clapham B., Brogan A.P., Dickerson T.J., Janda K.D. (2005). Revisiting quorum sensing: Discovery of additional chemical and biological functions for 3-oxo-*N*-acylhomoserine lactones. Proc. Natl. Acad. Sci. USA.

[98] Lehoux D.E., Sanschagrin F., Levesque R.C. (2000). Genomics of the 35-kb *pvd* locus and analysis of novel *pvdIJK* genes implicated in pyoverdine biosynthesis in *Pseudomonas aeruginosa*. FEMS Microbiol. Lett..

[99] Jimenez P.N., Koch G., Papaioannou E., Wahjudi M., Krzeslak J., Coenye T., Cool R.H., Quax W.J. (2010). Role of PvdQ in *Pseudomonas aeruginosa* virulence under iron-limiting conditions. Microbiology.

[100] Wang L., Zhang C., Gong F., Li H., Xie X., Xia C., Chen J., Song Y., Shen A., Song J. (2011). Influence of *Pseudomonas aeruginosa pvdQ* gene on altering antibiotic susceptibility under swarming conditions. Curr. Microbiol..

[101] Hannauer M., Schäfer M., Hoegy F., Gizzi P., Wehrung P., Mislin G.L., Budzikiewicz H., Schalk I.J. (2011). Biosynthesis of the pyoverdine siderophore of *Pseudomonas aeruginosa* involves precursors with a myristic or a myristoleic acid chain. FEBS Lett..

[102] Draganov D.I., Teiber J.F., Speelman A., Osawa Y., Sunahara R., La Du B.N. (2005). Human paraoxonases (PON1, PON2, and PON3) are lactonases with overlapping and distinct substrate specificities. J. Lipid Res..

[103] Camps J., Pujol I., Ballester F., Joven J., Simó J.M. (2011). Paraoxonases as potential antibiofilm agents: Their relationship with quorum-sensing signals in Gram-negative bacteria. Antimicrob. Agents. Chemother..

[104] Primo-Parmo S.L., Sorenson R.C., Teiber J., La Du B.N. (1996). The human serum paraoxonase/arylesterase gene (PON1) is one member of a multigene family. Genomics.

[105] Harel M., Brumshtein B., Meged R., Dvir H., Ravelli R.B., McCarthy A., Toker L., Silman I., Sussman J.L. (2007). 3-D structure of serum paraoxonase 1 sheds light on its activity, stability, solubility and crystallizability. Arh. Hig. Rada. Toksikol..

[106] Teiber J.F., Draganov D.I., La Du B.N. (2003). Lactonase and lactonizing activities of human serum paraoxonase (PON1) and rabbit serum PON3. Biochem. Pharmacol..

[107] Yang F., Wang L.H., Wang J., Dong Y.H., Hu J.Y., Zhang L.H. (2005). Quorum quenching enzyme activity is widely conserved in the sera of mammalian species. FEBS Lett..

[108] Ozer E.A., Pezzulo A., Shih D.M., Chun C., Furlong C., Lusis A.J., Greenberg E.P., Zabner J. (2005). Human and murine paraoxonase 1 are host modulators of *Pseudomonas aeruginosa* quorum-sensing. FEMS Microbiol. Lett..

[109] Stoltz D.A., Ozer E.A., Ng C.J., Yu J.M., Reddy S.T., Lusis A.J., Bourquard N., Parsek M.R., Zabner J., Shih D.M. (2007). Paraoxonase-2 deficiency enhances *Pseudomonas aeruginosa* quorum sensing in murine tracheal epithelia. Am. J. Physiol. Lung Cell. Mol. Physiol..

[110] Draganov D.I. (2010). Lactonases with organophosphatase activity: Structural and evolutionary perspectives. Chem. Biol. Interact..

[111] Furlong C.E., Suzuki S.M., Stevens R.C., Marsillach J., Richter R.J., Jarvik G.P., Checkoway H., Samii A., Costa L.G., Griffith A. (2010). Human PON1, a biomarker of risk of disease and exposure. Chem. Biol. Interact..

[112] Horke S., Witte I., Altenhöfer S., Wilgenbus P., Goldeck M., Förstermann U., Xiao J., Kramer G.L., Haines D.C., Chowdhary P.K. (2010). Paraoxonase 2 is down-regulated by the *Pseudomonas aeruginosa* quorum sensing signal *N*-(3-oxododecanoyl)-L-homoserine lactone and attenuates oxidative stress induced by pyocyanin. Biochem. J..

[113] Dong Y.H., Zhang X.F., Xu J.L., Zhang L.H. (2004). Insecticidal *Bacillus thuringiensis* silences *Erwinia carotovora* virulence by a new form of microbial antagonism, signal interference. Appl. Environ. Microbiol..

[114] Czajkowski R., Jafra S. (2009). Quenching of acyl-homoserine lactone-dependent quorum sensing by enzymatic disruption of signal molecules. Acta. Biochim. Pol..

[115] Zhou Y., Choi Y.L., Sun M., Yu Z. (2008). Novel roles of *Bacillus thuringiensis* to control plant diseases. Appl. Microbiol. Biotechnol..

[116] Jensen G.B., Hansen B.M., Eilenberg J., Mahillon J. (2003). The hidden lifestyles of *Bacillus cereus* and relatives. Environ. Microbiol..

[117] Chai Y., Tsai C.S., Cho H., Winans S.C. (2007). Reconstitution of the biochemical activities of the AttJ repressor and the AttK, AttL, and AttM catabolic enzymes of *Agrobacterium tumefaciens*. J. Bacteriol.

[118] Yeung A.T., Bains M., Hancock R.E. (2011). The sensor kinase CbrA is a global regulator that modulates metabolism, virulence, and antibiotic resistance in *Pseudomonas aeruginosa*. J. Bacteriol..

[119] Bodilis J., Barray S. (2006). Molecular evolution of the major outer-membrane protein gene (*oprF*) of *Pseudomonas*. Microbiology.

[120] Fito-Boncompte L., Chapalain A., Bouffartigues E., Chaker H., Lesouhaitier O., Gicquel G., Bazire A., Madi A., Connil N., Véron W. (2011). Full virulence of *Pseudomonas aeruginosa* requires OprF. Infect. Immun..

[121] Ochs M.M., McCusker M.P., Bains M., Hancock R.E. (1999). Negative regulation of the *Pseudomonas aeruginosa* outer membrane porin OprD selective for imipenem and basic amino acids. Antimicrob. Agents. Chemother..

[122] Hibbing M.E., Fuqua C., Parsek M.R., Peterson S.B. (2010). Bacterial competition: Surviving and thriving in the microbial jungle. Nat. Rev. Microbiol..

[123] Bassler B.L. (2002). Small talk. Cell-to-cell communication in bacteria. Cell.

[124] Riedel K., Hentzer M., Geisenberger O., Huber B., Steidle A., Wu H., Høiby N., Givskov M., Molin S., Eberl L. (2001). *N*-acylhomoserine-lactone-mediated communication between *Pseudomonas aeruginosa* and *Burkholderia cepacia* in mixed biofilms. Microbiology.

[125] Hooi D.S., Bycroft B.W., Chhabra S.R., Williams P., Pritchard D.I. (2004). Differential immune modulatory activity of *Pseudomonas aeruginosa* quorum-sensing signal molecules. Infect. Immun..

[126] Givskov M., de Nys R., Manefield M., Gram L., Maximilien R., Eberl L., Molin S., Steinberg P.D., Kjelleberg S. (1996). Eukaryotic interference with homoserine lactone-mediated prokaryotic signalling. J. Bacteriol..

[127] Moreau-Marquis S., Stanton B.A., O'Toole G.A. (2008). *Pseudomonas aeruginosa* biofilm formation in the cystic fibrosis airway. Pulm. Pharmacol. Ther..

[128] Donlan R.M. (2001). Biofilms and device-associated infections. Emerg. Infect. Dis..

[129] Taylor E., Webster T.J. (2011). Reducing infections through nanotechnology and nanoparticles. Int. J. Nanomed..

[130] Estrela A.B., Abraham W.-R. (2010). Combining biofilm-controlling compounds and antibiotics as a promising new way to control biofilm infections. Pharmaceuticals.

[131] Callow J.A., Callow M.E. (2011). Trends in the development of environmentally friendly fouling-resistant marine coatings. Nat. Commun..

[132] Schultz M.P., Bendick J.A., Holm E.R., Hertel W.M. (2011). Economic impact of biofouling on a naval surface ship. Biofouling.

[133] Yebra D. (2004). Antifouling technology—past, present and future steps towards efficient and environmentally friendly antifouling coatings. Prog. Org. Coat..

[134] Dobretsov S., Dahms H.U., Qian P.Y. (2006). Inhibition of biofouling by marine microorganisms and their metabolites. Biofouling.

[135] Fusetani N. (2011). Antifouling marine natural products. Nat. Prod. Rep..

[136] Dobretsov S., Teplitski M., Bayer M., Gunasekera S., Proksch P., Paul V.J. (2011). Inhibition of marine biofouling by bacterial quorum sensing inhibitors. Biofouling.

[137] Schumacher J.F., Aldred N., Callow M.E., Finlay J.A., Callow J.A., Clare A.S., Brennan A.B. (2007). Species-specific engineered antifouling topographies: Correlations between the settlement of algal zoospores and barnacle cyprids. Biofouling.

[138] Molino P.J., Childs S., Eason Hubbard M.R., Carey J.M., Burgman M.A., Wetherbee R. (2009). Development of the primary bacterial microfouling layer on antifouling and fouling release coatings in temperate and tropical environments in Eastern Australia. Biofouling.

[139] Wu J., Huang X. (2010). Use of ozonation to mitigate fouling in a long-term membrane bioreactor. Bioresour. Technol..

[140] Sarti P., Avigliano L., Görlach A., Brüne B. (2002). Superoxide and nitric oxide–participation in cell communication. Cell Death Differ..

[141] Herzberg M., Kang S., Elimelech M. (2009). Role of extracellular polymeric substances (EPS) in biofouling of reverse osmosis membranes. Environ. Sci. Technol..

[142] Liu C.X., Zhang D.R., He Y., Zhao X.S., Bai R. (2010). Modification of membrane surface for anti-biofouling performance: Effect of anti-adhesion and anti-bacteria approaches. J. Memb. Sci..

[143] Yeon K.M., Lee C.H., Kim J. (2009). Magnetic enzyme carrier for effective biofouling control in the membrane bioreactor based on enzymatic quorum quenching. Environ. Sct. Technol..

[144] Grigorakis K., Rigos G. (2011). Aquaculture effects on environmental and public welfare—The case of Mediterranean mariculture. Chemosphere.

[145] Skindersoe M.E., Ettinger-Epstein P., Rasmussen T.B., Bjarnsholt T., de Nys R., Givskov M. (2008). Quorum sensing antagonism from marine organisms. Mar. Biotechnol..

[146] Natrah F.M., Defoirdt T., Sorgeloos P., Bossier P. (2011). Disruption of bacterial cell-to-cell communication by marine organisms and its relevance to aquaculture. Mar. Biotechnol..

[147] Verschuere L., Rombaut G., Sorgeloos P., Verstraete W. (2000). Probiotic bacteria as biological control agents in aquaculture. Microbiol. Mol. Biol. Rev..

[148] Balcázar J.L., Rojas-Luna T., Cunningham D.P. (2007). Effect of the addition of four potential probiotic strains on the survival of pacific white shrimp (*Litopenaeus vannamei*) following immersion challenge with *Vibrio parahaemolyticus*. J. Invertebr. Pathol..

[149] Nhan D.T., Cam D.T., Wille M., Defoirdt T., Bossier P., Sorgeloos P. (2010). Quorum quenching bacteria protect *Macrobrachium rosenbergii* larvae from *Vibrio harveyi* infection. J. Appl. Microbiol..

[150] Manefield M., Harris L., Rice S.A., de Nys R., Kjelleberg S. (2000). Inhibition of luminescence and virulence in the black tiger prawn (*Penaeus monodon*) pathogen *Vibrio harveyi* by intercellular signal antagonists. Appl. Environ. Microbiol..

[151] Rasch M., Buch C., Austin B., Slierendrecht W.J., Ekmann K.S., Larsen J.L., Johansen C., Riedel K., Eberl L., Givskov M. (2004). An inhibitor of bacterial quorum sensing reduces mortalities caused by Vibriosis in rainbow trout (*Oncorhynchus mykiss, Walbaum)*. Syst. Appl. Microbiol..

[152] Plough H.H. (1945). Penicillin resistance of *Staphylococcus aureus* and its clinical implications. Am. J. Clin. Pathol..

[153] Demain A.L., Sanchez S. (2009). Microbial drug discovery: 80 years of progress. J. Antibiot. (Tokyo).

[154] Rasko D.A., Sperandio V. (2010). Anti-virulence strategies to combat bacteria-mediated disease. Nat. Rev. Drug Discov..

[155] Rasmussen T.B., Givskov M. (2006). Quorum-sensing inhibitors as anti-pathogenic drugs. Int. J. Med. Microbiol..

[156] Bjarnsholt T., Givskov M. (2007). Quorum-sensing blockade as a strategy for enhancing host defences against bacterial pathogens. Philos. Trans. R Soc. Lond. B Biol. Sci..

[157] Dobretsov S., Teplitski M., Paul V. (2009). Mini-review: Quorum sensing in the marine environment and its relationship to biofouling. Biofouling.

[158] Ni N., Li M., Wang J., Wang B. (2009). Inhibitors and antagonists of bacterial quorum sensing. Med. Res. Rev..

[159] Defoirdt T., Boon N., Bossier P. (2010). Can bacteria evolve resistance to quorum sensing disruption?. PLoS Pathog..

[160] Köhler T., Perron G.G., Buckling A., van Delden C. (2010). Quorum sensing inhibition selects for virulence and cooperation in *Pseudomonas aeruginosa*. PLoS Pathog..

[161] Maeda T., García-Contreras R., Pu M., Sheng L., Garcia L.R., Tomás M., Wood T.K. (2012). Quorum quenching quandary: Resistance to antivirulence compounds. ISME J..

[162] Poole K., Tetro K., Zhao Q., Neshat S., Heinrichs D.E., Bianco N. (1996). Expression of the multidrug resistance operon *mexA-mexB-oprM* in *Pseudomonas aeruginosa: mexR* encodes a regulator of operon expression. Antimicrob. Agents Chemother..

[163] Cao L., Srikumar R., Poole K. (2004). MexAB-OprM hyperexpression in NalC-type multidrug-resistant *Pseudomonas aeruginosa*: Identification and characterization of the *nalC* gene encoding a repressor of PA3720-PA3719. Mol. Microbiol..

[164] Daigle D.M., Cao L., Fraud S., Wilke M.S., Pacey A., Klinoski R., Strynadka N.C., Dean C.R., Poole K. (2007). Protein modulator of multidrug efflux gene expression in *Pseudomonas aeruginosa*. J. Bacteriol..

[165] Gotschlich A., Huber B., Geisenberger O., Tögl A., Steidle A., Riedel K., Hill P., Tümmler B., Vandamme P., Middleton B. (2001). Synthesis of multiple *N*-acylhomoserine lactones is wide-spread among the members of the *Burkholderia cepacia* complex. Syst. Appl. Microbiol..

[166] Zhu J., Beaber J.W., Moré M.I., Fuqua C., Eberhard A., Winans S.C. (1998). Analogs of the autoinducer 3-oxooctanoyl-homoserine lactone strongly inhibit activity of the TraR protein of *Agrobacterium tumefaciens*. J. Bacteriol..

[167] Brader G., Sjöblom S., Hyytiäinen H., Sims-Huopaniemi K., Palva E.T. (2005). Altering substrate chain length specificity of an acylhomoserine lactone synthase in bacterial communication. J. Biol. Chem..

[168] Case R.J., Labbate M., Kjelleberg S. (2008). AHL-driven quorum-sensing circuits: Their frequency and function among the Proteobacteria. ISME J..

[169] Patankar A.V., González J.E. (2009). Orphan LuxR regulators of quorum sensing. FEMS Microbiol. Rev..

[170] Van Delden C., Pesci E.C., Pearson J.P., Iglewski B.H. (1998). Starvation selection restores elastase and rhamnolipid production in a *Pseudomonas aeruginosa* quorum-sensing mutant. Infect. Immun..

[171] Sandoz K.M., Mitzimberg S.M., Schuster M. (2007). Social cheating in *Pseudomonas aeruginosa* quorum sensing. Proc. Natl. Acad. Sci. USA.

[172] Beatson S.A., Whitchurch C.B., Semmler A.B., Mattick J.S. (2002). Quorum sensing is not required for twitching motility in *Pseudomonas aeruginosa*. J. Bacteriol..

[173] Dekimpe V., Déziel E. (2009). Revisiting the quorum-sensing hierarchy in *Pseudomonas aeruginosa*: The transcriptional regulator RhlR regulates LasR-specific factors. Microbiology.

[174] Choi Y., Park H.Y., Park S.J., Park S.J., Kim S.K., Ha C., Im S.J., Lee J.H. (2011). Growth phase-differential quorum sensing regulation of anthranilate metabolism in *Pseudomonas aeruginosa*. Mol. Cells.

[175] Bardill J.P., Zhao X., Hammer B.K. (2011). The *Vibrio cholerae* quorum sensing response is mediated by Hfq-dependent sRNA/mRNA base pairing interactions. Mol. Microbiol..

[176] Fuqua W.C., Winans S.C. (1994). A LuxR-LuxI type regulatory system activates *Agrobacterium* Ti plasmid conjugal transfer in the presence of a plant tumor metabolite. J. Bacteriol..

[177] Wei J.R., Tsai Y.H., Horng Y.T., Soo P.C., Hsieh S.C., Hsueh P.R., Horng J.T., Williams P., Lai H.C. (2006). A mobile quorum-sensing system in *Serratia marcescens*. J. Bacteriol..

[178] Imamura Y., Yanagihara K., Tomono K., Ohno H., Higashiyama Y., Miyazaki Y., Hirakata Y., Mizuta Y., Kadota J., Tsukamoto K. (2005). Role of *Pseudomonas aeruginosa* quorum-sensing systems in a mouse model of chronic respiratory infection. J. Med. Microbiol..

[179] Lesic B., Lépine F., Déziel E., Zhang J., Zhang Q., Padfield K., Castonguay M.H., Milot S., Stachel S., Tzika A.A. (2007). Inhibitors of pathogen intercellular signals as selective anti-infective compounds. PLoS Pathog..

[180] Rainey P.B., Rainey K. (2003). Evolution of cooperation and conflict in experimental bacterial populations. Nature.

[181] Diggle S.P., Griffin A.S., Campbell G.S., West S.A. (2007). Cooperation and conflict in quorum-sensing bacterial populations. Nature.

[182] Mellbye B., Schuster M. (2011). The sociomicrobiology of antivirulence drug resistance: A proof of concept. mBio.

[183] Clatworthy A.E., Pierson E., Hung D.T. (2007). Targeting virulence: A new paradigm for antimicrobial therapy. Nat. Chem. Biol..

